# Minor stroke patients with mild-moderate diastolic blood pressure derive greater benefit from dual antiplatelet therapy

**DOI:** 10.1038/s41440-023-01422-8

**Published:** 2023-09-05

**Authors:** Tingting Liu, Yongle Wang, Yanan Li, Kaili Zhang, Haimei Fan, Jing Ren, Juan Li, Yali Li, Xinyi Li, Xuemei Wu, Junhui Wang, Lixi Xue, Xiaolei Gao, Yuping Yan, Gaimei Li, Qingping Liu, Wenhua Niu, Wenxian Du, Yuting Liu, Xiaoyuan Niu

**Affiliations:** 1 Department of Neurology, First Hospital of Shanxi Medical University, No. 85, Jiefang Nan Street, Yingze District, Taiyuan, Shanxi China; 2 Shanxi Medical University, No. 58, Xinjian Nan Street, Yingze District, Taiyuan, China; 3 Department of Neurology of Shanxi Bethune Hospital, Shanxi Academy of Medical Sciences, Tongji Shanxi Hospital, Third Hospital of Shanxi Medical University, Taiyuan, 030032 China; 4 Department of Neurology, Sixth Hospital of Shanxi Medical University (General Hospital of Tisco), Taiyuan, Shanxi China; 5 Shanxi Province Cardiovascular Disease Hospital, Taiyuan, Shanxi China; 6 Chanzhou Central Hospital, Chanzhou, Hebei China; 7 Yanhu Branch First Hospital of Shanxi Medical University, Yuncheng, Shanxi China; 8 Taiyuan Wanbailin District Medical Group Central Hospital, Taiyuan, Shanxi China; 9 China Railway 17th Bureau Group Company Central Hospital, Taiyuan, Shanxi China; 10 First People’s Hospital of JIN ZHONG, Jinzhong, Shanxi China

**Keywords:** Dual antiplatelet therapy, Diastolic blood pressure, Minor stroke

## Abstract

Not only systolic blood pressure (SBP) but also diastolic blood pressure (DBP) increases the risk of recurrence in the short- or long-term outcomes of stroke. The interaction between DBP and antiplatelet treatment for China stroke patients is unclear. This multicenter, observational cohort study included 2976 minor ischemic stroke patients. Patients accepted single antiplatelet therapy (SAPT) or dual antiplatelet therapy (DAPT) after arrival, and baseline DBP levels were trichotomized into <90 mmHg, 90–110 mmHg and ≥110 mmHg. We explore the interaction effect between antiplatelet therapy and DBP on 90-days composite vascular events. A total of 257 (8.6%) patients reached a composite vascular event during follow-up. The interaction term between DBP levels and treatment group (SAPT vs. DAPT) was significant (*P* for interaction = 0.013). DAPT’s adjusted HR for composite events in patients with DBP between 90 and 110 mmHg was 0.56 (95% confidence interval, 0.36 0.88; *P* = 0.011) and DBP ≥ 110 mmHg was 4.35 (95% confidence interval, 1.11–19.94; *P* = 0.046). The association between treatment and DBP was still consistent after propensity score matching of the baseline characteristics. The interaction term of DBP ×  treatment was not significant for the safety outcomes of severe bleeding (*P* for interaction = 0.301) or hemorrhage stroke (*P* for interaction = 0.831). In this cohort study based on the real world, patients with a DBP between 90 and 110 mmHg received a greater benefit from 90 days of DAPT than those with lower and higher baseline DBP. REGISTRATION: (https://www.chictr.org.cn; Unique identifier: ChiCTR1900025214)

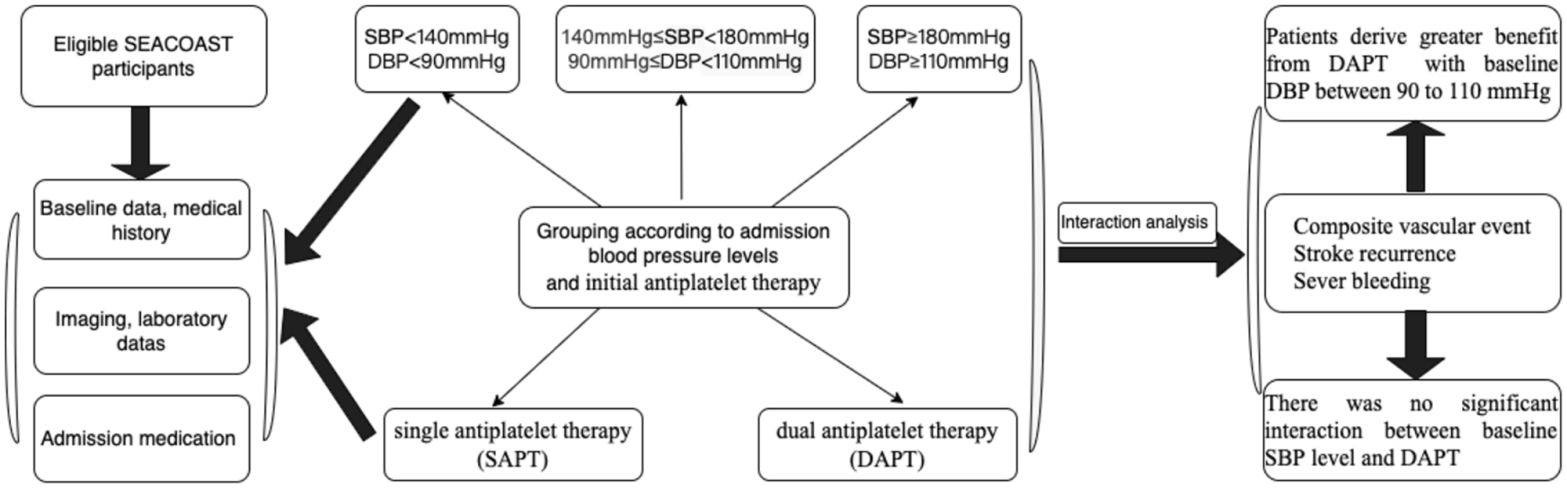

## Introduction

Approximately 75% of patients who have an acute ischemic stroke (AIS) experience elevated blood pressure (BP) [1, 2]. The International Stroke Test [3] revealed that 81.6% of patients had elevated systolic BP (SBP ≥ 140 mmHg), and 30.5% had elevated diastolic BP (DBP ≥ 90 mmHg) within 48 h after stroke. In patients with compromised cerebral circulation of ischemic tissue, elevated BP is viewed as a pathophysiological response to ensure adequate cerebral perfusion [4]. Previous studies suggest that acute hypertensive response is an independent prognostic factor for stroke, especially systolic BP rather than diastolic BP. However, an analysis of data from 1.3 million outpatient clinics found that systolic and diastolic BP independently affected the prognosis of adverse cardiovascular events [5]. Intensive reduction of DBP may lead to persistent subclinical myocardial injury, impaired perfusion, and autoregulation of vital organs, which may lead to worse cardiovascular and cerebrovascular outcomes. Management of diastolic BP during the acute phase of stroke is gaining increasing attention among physicians.

The relationship between BP level and cardiovascular and cerebrovascular outcomes is still highly controversial. Most hypertension studies have found a curved relationship between BP level and the occurrence of cardiovascular and cerebrovascular events. SBP is called a U-shaped curve, and DBP is a J-shaped or U-shaped curve [6]. In the Systolic BP Intervention Trial (SPRINT), baseline SBP had a U-shaped relationship with all-cause death, and baseline DBP had a U-shaped association with the hazard of cerebrovascular disease [7]. Several studies have confirmed that BP levels at admission may interact with antiplatelet regimens on stroke prognosis. The Clopidogrel with Aspirin in Acute Minor Stroke or Transient Ischemic Attack (CHANCE) trial subgroup analysis found that patients with SBP above 140 mmHg or DBP above 90 mmHg benefited more from DAPT [8]. However, it did not report the interaction effect. The Platelet-Oriented Inhibition in New TIA and Minor Ischemic Stroke (POINT) trial found that patients with SBP below 140 mmHg benefited more from DAPT and that there was an interaction relationship between SBP and the treatment regimen [9]. The basic hypothesis of the above study designs is that both BP and platelet reactivity will influence endothelial cell stability, and as a result, different levels of BP may amplify or reduce the efficacy of antiplatelet therapy. However, few reports have demonstrated the relationship between diastolic pressure and antiplatelet treatment in the management of AIS patients. Therefore, the study aims to investigate whether there is also an interaction relationship between BP and antiplatelet treatment in the real world.

Point of view

- Clinical relevance
  Baseline BP levels during the acute period are associated with stroke prognosis, but the impact of baseline BP on the efficacy of DAPT is inconsistent.
- Future direction
  It is necessary to conduct larger population-based experiments to validate the correlation between acute verification BP and the effectiveness of DAPT, especially the potential impact of acute BP variability on the effectiveness of DAPT.
- Consideration for the Asian population
  Different populations may benefit to varying degrees from DAPT at different baseline BP levels, and for Asian populations, a slightly higher BP level during the acute period may be more beneficial for ischemic stroke patients receiving DAPT.

## Methods

### Study participants and design

The study analysis is based on a multicenter, prospective observational real-world database of minor ischemic stroke patients admitted to 8 stroke centers in Shanxi Province, China. The article was constructed according to the guideline of STROBE in Epidemiology [10]. Detailed information about the database is provided in the Supplement. The dataset included minor ischemic stroke patients with National Institute of Health stroke scale (NIHSS) scores ≤5 points within 72 h of symptom onset. Higher scores on the NIHSS, which varied from 0 to 42, indicated more severe stroke. Patients were admitted between September 2019 and November 2021. The inclusion criteria included (1) acute minor ischemic stroke (defined as National Institutes of Health Stroke Scale ≤5), (2) within 72 h of symptom onset (symptom onset is defined by the “last seen as normal”), and (3) treatment with antiplatelet drugs. Exclusion criteria were provided in the Supplement protocol. According to the initial antiplatelet regimen used after arrival, study subjects were divided into two groups: single antiplatelet therapy (SAPT) with monotherapy of aspirin (dose of 81 mg/100 mg/200 mg/300 mg) or clopidogrel (dose of 75 mg/150 mg/300 mg) and dual antiplatelet therapy (DAPT) with aspirin (dose of 81 mg/100 mg/200 mg/300 mg) plus clopidogrel (load dose of 75 mg/150 mg/300 mg).

### Data collection

Data are currently registered in a web-based registry available at https://www.palacetrial.cn, based on Research Electronic Data Capture, a secure web-based software platform that supports data capture for research studies [11]. All researchers were trained in a unified manner before data collection. Clinical information was prospectively collected from eight stroke centers in Shanxi Province. Ten trained neurologists collected the data from the electronic medical department. Demographic: including age, sex, body mass index(BMI), admission SBP and DBP level, smoking, history of diseases and medical history; clinical evaluation: including the time from onset to arrival (categorized as ≤24 h and among 24 h to 72 h), initial NIHSS scores (categorized as ≤3 score and between 4 and 5 score), pre-stroke mRs score, and ischemic stroke subtype according to the TOAST criteria; imaging information: including eligible CT and MRI scans and vascular examination (TCD/MRA/CTA/DSA); laboratory data: including white blood cell counts, creatinine serum levels, platelet counts, international normalized ratio, urea, homocysteine (HCY), and fasting low-density lipoprotein cholesterol (LDL-C); in-hospital treatments: including antiplatelet (including single antiplatelet therapy of aspirin or clopidogrel, dual antiplatelet therapy of aspirin plus clopidogrel); and some indicators are monitored, including daily BP measurements.

### Blood pressure measurement

Trained nurses measured the BP of patients resting in the supine position using an automated cuff, mercury, or aneroid sphygmomanometer. We divided patients according to admission SBP levels (<140 mmHg, 140–180 mmHg, and ≥180 mmHg) and admission DBP levels (<90 mmHg, 90–110 mmHg, and ≥110 mmHg). Other BP measurements were obtained twice a day within 3 days after admission: from 10:00 am to 02:00 pm and from 06:00 pm to 10:00 pm. BP values with ≥5 measurements were used to calculate the variability indices. Intraindividual reading-to-reading systolic or diastolic BP variability was evaluated by two indices, including the standard deviation (SD) of BP and the coefficient of variation (CV) of BP (SD/mean × 100). Means and peaks SBP and DBP were also calculated for each patient.

### Outcomes

To investigate whether DAPT treatment and stroke risk differed by diastolic blood pressure, we developed a composite vascular event (ischemic stroke recurrence, TIA, symptomatic intracerebral hemorrhage, myocardial infarction or angina attacks, and vascular death) as the primary outcome at 90 days. Secondary outcomes were each component of the primary outcome. Definition of secondary outcomes provided in the supplement protocol. Safety endpoints included severe bleeding (GUSTO criteria, Global Use of Strategies to Open Occluded Coronary Arteries definition) [12], hemorrhagic stroke, and all bleeding events during follow-up. Each patient was contacted for follow-up at 90 days after symptom onset via telephone by trained researchers. A set of uniform structured questionnaires was used by trained personnel to ensure the accuracy of the interviews.

### Statistical analysis

Statistical analysis was performed based on a modified intention to treat (ITT) principle. After stratification by DBP, patient characteristics were summarized. The frequency (percentage) is reported, and the mean ± standard deviation or the median (interquartile interval) is reported. We tested for intergroup differences with the *t* test for continuous variables and the χ^2^ test or Fisher’s exact probability test for categorical variables. Univariate ANOVA was used to compare continuous variables for baseline data, and Least Significant Difference method was used for multicombination comparison. Our outcomes are fitted with Cox proportional hazards models and the adjusted and unadjusted hazard ratios are reported. BP levels, history of hypertension, past use of antihypertension drugs and age were analyzed for interaction with antiplatelet treatment on the outcomes. Stratified Cox models by SBP and DBP levels were performed and the hazard ratios for different outcomes were reported in the stratifications. A Kaplan-Meier failure curve was used to illustrate the distribution of events in the stratified groups, and the log-rank test was used to generate *P* values. A rigorous adjustment for differences in characteristics of patients was performed by propensity score matching, using inverse probability of treatment weighting, to control selection biases and to determine the causal effect of antiplatelet treatment on outcomes. Throughout, 2-tailed tests were used with statistical significance defined as *P* < 0.05. All analyses were performed in SPSS 26.0 and the statistical software R 4.2.1 (http://www.R-project.org, The R Foundation).

## Results

### Baseline characteristics

**Fig. 1:**
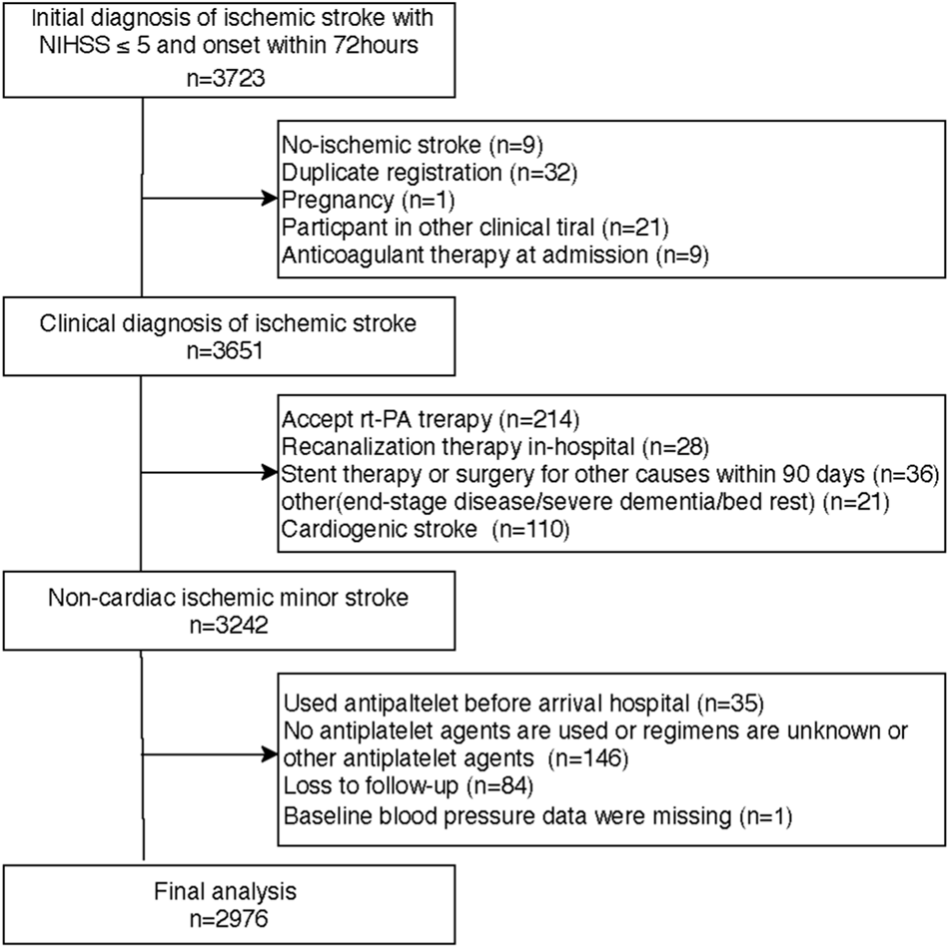
Flow chart of patient inclusion and exclusion

A total of 3723 eligible and 747 patients were excluded. The detailed reasons for exclusion are described in Fig. 1. Finally, 2976 patients were included in the analysis. There were 1163 (39.1%) patients in the SAPT group and 1813 (60.9%) patients in the DAPT group. The mean (SD) age was 61.7 ± 11.9 years; 2182 (73.3%) were male individuals. Based on DBP level, Table 1 shows the baseline demographic characteristics (<90 mmHg vs. 90–110 mmHg vs. ≥ 110 mmHg). There were 1639 patients (55.1%) with a DBP less than 90 mmHg, 1144 patients (38.4%) with a DBP greater than or equal to 90 mmHg and fewer than 110 mmHg and 193 patients (6.5%) with a DBP greater than 110 mmHg, as shown in Table 1. Patient characteristics were significantly different in age, sex, BMI, smoking status, history of HTN, DM, ischemic stroke, previous antiplatelet, antihypertension and statin drug use, baseline NIHSS, prestr-oke mRs, TOAST, LDL-C, HCY level. Supplementary Tables 1 and 2 show baseline demographic characteristics stratified by initial antiplatelet drugs and admission SBP levels.

**Table 1:** Baseline characteristic of patients stratified by diastolic blood pressure

Variables/DBP	<90 mmHg (*n* = 1639)	90–110 mmHg (*n* = 1144)	≥110 mmHg (*n* = 193)	*P* value
Age, years	63.8 ± 12.1	59.9 ± 11.0^a^	54.6 ± 11.7^b^	<0.001
Sex, *n* (%)				<0.001
Female	479 (29.2)	284 (24.8)^a^	31 (16.1)^b^	
Male	1160 (70.8)	860 (75.2)	162 (83.9)	
BMI, kg/m^2^	24.6 ± 3.6	25.1 ± 3.3^a^	26.2 ± 3.8^b^	<0.001
Systolic pressure, mmHg	143.2 ± 18.9	162.2 ± 18.0^a^	180.0 ± 19.6^b^	<0.001
Diastolic pressure, mmHg	78.5 ± 7.7	97.9 ± 5.6^a^	116.4 ± 6.9^b^	<0.001
Smoking status, no. (%)				0.011
Never	812 (49.8)	536 (47.0)	72 (38.5)	
Previous smoking	106 (6.5)	66 (5.8)	10 (5.3)	
Current smoking	681 (41.8)	505 (44.3)^a^	99 (52.9)^b^	
Medical history, no. (%)				
Hypertension	897 (54.7)	759 (66.3)^a^	147 (76.2)^b^	<0.001
Diabetes mellitus	478 (29.2)	286 (25.0)^a^	27 (14.0)^b^	<0.001
Lipid disorder	34 (2.1)	29 (2.5)	6 (3.1)	0.461
AF	9 (0.5)	5 (0.4)	2 (1.0)	0.411
TIA	31 (1.9)	17 (1.5)	1 (0.5)	0.361
IS	400 (24.4)	244 (21.3)^a^	32 (16.5)^b^	0.018
PAD	15 (0.9)	9 (0.8)	0 (0)	0.544
AM	31 (1.9)	14 (1.2)	3 (1.6)	0.387
CAD	103 (6.3)	54 (4.7)	8 (3.6)	0.102
ICH	31 (1.9)	29 (2.5)	4 (2.1)	0.497
Medication history use, *n* (%)				
Antiplatelet	206 (12.6)	120 (10.5)^a^	9 (4.7)^b^	0.006
Anticoagulated	3 (0.2)	0 (0)	0 (0)	0.664
Antihypertensive	633 (38.6)	506 (44.2)^a^	90 (46.6)^b^	<0.001
Statins	141 (8.6)	80 (7.0)	2 (1.0)^b^	<0.001
Medication onset, *n* (%)				
Antiplatelet				<0.001
SAPT	701 (42.8)	393 (34.4)^a^	69 (35.8)^b^	
DAPT	938 (57.2)	751 (65.6)^a^	124 (64.2)^b^	
Statin	1627 (99.3)	1136 (99.3)	192 (99.5)	1.000
Duration of DAPT, days	35.0 ± 32.6	33.2 ± 32.0	32.5 ± 30.7	0.434
Clinical evaluation, no. (%)				
Baseline NIHSS score				0.013
≤3	1355 (82.7)	911 (79.6)	145 (75.1)^b^	
4–5	284 (17.3)	233 (20.4)	48 (24.9)^b^	
Onset to arrival time				0.688
≤24 hours	909 (55.5)	649 (56.7)	112 (58.0)	
24–72 hours	730 (44.5)	495 (43.3)	81 (42.0)	
Pre-stroke mRS				0.006
0	1380 (84.2)	974 (85.1)	179 (93.2)^b^	
1	220 (13.4)	139 (12.2)	9 (4.7)^b^	
2	39 (2.4)	31 (2.7)	4 (2.1)	
TOAST				0.0005
LAA	515 (31.4)	342 (29.9)	41 (21.1)^b^	
SVO	724 (44.2)	537 (47.0)	107 (55.4)^b^	
OE/UD	400 (24.4)	264 (23.1)	45 (23.2)	
ICAS^c^				0.442
No	942 (57.5)	629 (55.0)	117 (60.6)	
Yes	640 (39.0)	471 (41.2)	71 (36.8)	
Laboratory results (Mean ± SD)				
LDL-C, mmol/L	2.6 ± 0.8	2.6 ± 0.8	2.7 ± 0.9^b^	0.005
HCY, μmol/L	23.2 ± 20.4	24.4 ± 22.1	28.1 ± 24.5^b^	0.014
Creatinine, μmol/L	74.5 ± 41.6	74.3 ± 28.8	78.8 ± 31.6	0.312
Urea, mmol/L	5.8 ± 15.9	5.3 ± 2.1^a^	5.2 ± 1.8	0.495
INR	1.1 ± 0.3	1.1 ± 0.4	1.1 ± 0.3	0.713
WBC, 10^9^/L	7.1 ± 2.3	7.2 ± 2.1	7.4 ± 2.2	0.200
PLT, 10^9^/L	215.8 ± 69.1	219.7 ± 61.0	214.1 ± 57.5	0.276

*AF* atrial fibrillation, *CAD* coronary artery disease, *DM* diabetes mellitus, *HTN* hypertension, *ICAS* intracranial cerebral atherosclerosis, *ICH* intracranial hemorrhage, *LAA* large artery atherosclerosis, *LDL-C* low-density lipoprotein cholesterol, *mRS* modified rankin scale, *NIHSS* national institutes of health stroke scale, *OE* other etiology, *PAD* peripheral artery disease, *SVO* small vessel occlusion, *TIA* transient ischemic attack, *TOAST* trial of ORG 10172 in acute stroke treatment, *UD* undetermined etiology, *WBC* white blood cell counts

^a^Indicate there were statistical differences between the two groups of DBP between 90 and 110 mmHg and DBP < 90 mmHg

^b^Indicate there were statistical differences between the two groups of DBP ≥ 110 mmHg and DBP < 90 mmHg

^c^Indicate the patients complete vascular imaging data

### Outcomes

**Fig. 2:**
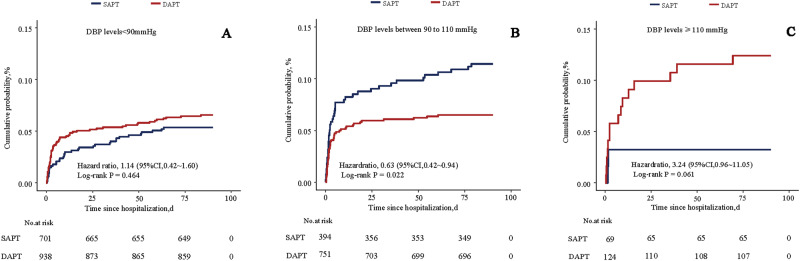
Cumulative probability of primary events according to treatment and baseline DBP groups. **A** patients with DBP < 90 mmHg; **B** patients with DBP between 90 and 110 mmHg; **C** patients with DBP ≥ 110 mmHg

257 patients (8.6%) achieved the primary outcome of composite vascular events during follow-up. The event rate of ischemic stroke in patients with DBP less than 90 mmHg for SAPT group was 8.0% (56 of 701) and DAPT group was 8.6% (81 of 938), whereas in patients with DBP greater than or equal to 90 mmHg and less than 110 mmHg, it was 11.2% (44 of 393) vs. 7.5% (56 of 751), and in patients with DBP more than 110 mmHg, it was 4.3% (3 of 69) vs. 13.7% (17 of 124). The main adjusted Cox model fit to the primary outcome met the proportional hazards assumption. The interaction term between DBP (<90 mmHg vs. 90–110 mmHg vs. ≥110 mmHg) and treatment group (SAPT vs. DAPT) was significant in the unadjusted (*P* for interaction = 0.006) and adjusted (*P* for interaction = 0.013) models. After stratification by DBP, DAPT’s adjusted hazard ratio (HR) for all stroke in patients with DBP less than 90 mmHg was 1.35 (95% CI, 0.92–1.96, *P* = 0.122), whereas in those with DBP greater than or equal to 90 mmHg and less than 110 mmHg, the HR was 0.56 (95% CI, 0.36 0.88; *P* = 0.011), and in those with DBP greater than or equal to 110 mmHg, the HR was 4.35 (95% CI, 1.11–19.94; *P* = 0.046). These differences are illustrated in the Kaplan–Meier curves in Fig. 2, which show the reduction in early composite vascular events seen in patients with DBP greater than or equal to 90 mmHg and less than 110 mmHg on DAPT. A similar association was seen for all stroke and ischemic stroke outcomes (Table 2). To further verify the accuracy of the results, all the data were matched with propensity scores to control potential selection biases between SAPT and DAPT (Supplementary Table 3). The results still showed a consistent interaction (P for interaction = 0.009), and the benefit from DAPT was more pronounced in patients with diastolic BP from 90 to 110 mmHg (HR = 0.55 (95% CI, 0.34–0.88), *P* = 0.026). Patients with diastolic blood pressures below 90 mmHg or greater than or equal to 110 mmHg did not appear to benefit from DAPT. No statistically significant interaction was found between SBP (stratified as less than 140 mmHg, 140–180 mmHg, and equal to or greater than 180 mmHg) and antiplatelet treatment (DAPT vs. SAPT) on the primary outcome (*P* for interaction = 0.719), consistent with the matched data (Supplementary Table 4 and Supplementary Fig. 4). Patients with an SBP greater than 180 mmHg had the highest primary event rate during follow-up, though there was no significant association between DAPT treatment and composite vascular events (HR for composite vascular events, 1.39; 95% CI, 0.69–2.81; *P* = 0.280). There was no significant interaction between the two items (history of hypertension and past use of antihypertension drugs) and antiplatelet treatment for primary outcomes (Supplementary Fig. 1). A further stratification analysis based on age and DBP levels was also performed. A significant interaction effect was observed between age × DBP and antiplatelet treatment on composite vascular events. Only patients over 55 years old, with DBP from 90 to 110 mmHg can benefit from DAPT to reduce risk of composite vascular events (HR = 0.57 (95% CI, 0.36–0.91), *P* = 0.020) (Supplementary Fig. 2).

**Table 2:** Efficacy Outcomes of Patients with Different Antiplatelet Therapies Stratified by baseline DBP and the Model with the Interaction Term of DBP and treatment

Outcome	SAPT event, no. (%)/total no.	DAPT event, no. (%)/total no.	Crude HR (95%CI)	Crude *P* value	Adj HR* (95%CI)	Adj *P* value	*P* for interaction
DBP level, mmHg							
Primary outcome							
Composite vascular events							
<90	56/701 (8.0)	81/938 (8.6)	1.14 (0.81–1.60)	0.464	1.35 (0.92–1.96)	0.122	0.013
90–110	44/393 (11.2)	56/751 (7.5)	0.63 (0.42–0.94)	0.022	0.56 (0.36–0.88)	0.011	
≥110	3/69 (4.3)	17/124 (13.7)	3.24 (0.96–11.05)	0.061	4.35 (1.11–19.94)	0.046	
Secondary outcomes							
All stroke							
<90	53/701 (7.6)	76/938 (8.1)	1.15 (0.80–1.64)	0.444	1.31 (0.89–1.93)	0.174	0.025
90–110	43/393 (10.2)	56/751 (7.2)	0.64(0.43–0.96)	0.031	0.58 (0.37–0.92)	0.020	
≥110	3/69 (4.3)	16/124 (12.9)	3.014(0.88–10.35)	0.077	5.01 (1.21–22.52)	0.035	
Ischemic stroke							
<90	45/701 (6.4)	69/938 (7.4)	1.21 (0.83–1.77)	0.323	1.37 (0.91–2.07)	0.135	0.034
90–110	40/393 (10.2)	54/751 (7.2)	0.67 (0.44–1.01)	0.054	0.61 (0.38–0.97)	0.035	
≥110	3/69 (4.3)	14/124 (11.3)	2.64 (0.75–9.1)	0.131	4.10 (1.03–18.25)	0.064	
TIA							
<90	5/701 (0.7)	6/938 (0.6)	0.9 (0.27–1.68)	0.857	0.96 (0.24–3.79)	0.953	0.104
90–110	3/393 (0.8)	1/751 (0.1)	0.17 (0.02–1.68)	0.131	0 (0–Inf)	0.999	
≥110	0/69 (0)	2/124 (1.6)	Inf	0.999	13.91 (0–Inf)	1	
Symptomatic intracerebral hemorrhage							
<90	3/701 (0.4)	1/938 (0.1)	0.37 (0.03–4.11)	0.420	0.39 (0.02–4.77)	0.471	0.301
90–110	0/393 (0)	1/751 (0.1)	Inf	1	Inf	1	
≥110	0/69 (0)	0/124 (0)	1 (1–1)	-	1 (1–1)	-	
Myocardial infarction or angina attacks							
<90	1/701 (0.1)	4/938 (0.4)	2.98 (0.33–26.71)	0.328	4.83(0.38–60.9)	0.223	0.153
90–110	1/393 (0.3)	0/751 (0)	0 (0–Inf)	0.999	0 (0–Inf)	1	
≥110	0/69 (0)	1/124 (0.8)	Inf	0.999	0 (0–Inf)	NaN00	
Vascular death							
<90	2/701 (0.3)	1/938 (0.1)	0.37 (0.03–4.11)	0.420	0.75(0.04–15.78)	0.852	1
90–110	0/393 (0)	0/751 (0)	1 (1–1)	-	1 (1–1)	-	
≥110	0/69 (0)	0/124 (0)	1 (1–1)	-	1 (1–1)	-	

*Adjusted for factors of sex, age, BMI, baseline NIHSS, onset time to hospital arrival, statin use at admission, smoking, previous IS, ICH, AF, creatinine, ICAS, TOAST

### Safety outcome

Severe bleeding, as defined according to the GUSTO criteria (the primary safety outcome event), occurred in three patients (0.3%) in the SAPT group and two patients (0.1%) in the DAPT group (HR 0.62; 95% CI, 0.09 to 4.4; *P* = 0.632). Hemorrhagic stroke occurred in 30 patients (2.6%) in the SAPT group and 33 patients in the DAPT group (1.8%). The interaction term of DBP × treatment was not significant for the safety outcomes of severe bleeding (*P* for interaction = 0.301) or hemorrhage stroke (*P* for interaction = 0.831) (Table 3). The rate of hemorrhagic stroke in the DAPT group was 2.0% (10 of 488) in the group with a SBP less than 140 mmHg, 1.6% (18 of 1112) in the group it a SBP between 140 and 180 mmHg, and 2.3% (5 of 213) in the group with a SBP equal to or greater than 180 mmHg. The interaction term of SBP × treatment was not significant for the safety outcomes of severe bleeding (*P* for interaction = 0.520) or hemorrhage stroke (*P* for interaction = 0.580) (Supplementary Table 5).

**Table 3:** Safety Outcomes of Patients with Different Antiplatelet Therapies Stratified by baseline DBP and the Model with the Interaction Term of DBP and treatment

Outcome	SAPT event, no. (%)/total no.	DAPT event, no. (%)/total no.	Crude HR (95%CI)	Crude *P* value	Adj HR (95%CI)	Adj *P* value	*P* for interaction
DBP level, mmHg							
Safety outcomes							
Severe bleeding							
<90	3/701 (0.4)	1/938 (0.1)	0.37 (0.03–4.11)	0.420	0.39 (0.02–5.09)	0.471	0.301
90–110	0/393 (0)	1/751 (0.1)	Inf (0–Inf)	0.999	Inf (0–Inf)	1.0	
≥110	0/69 (0)	0/124 (0)	1 (1–1)	-	1 (1–1)	-	
Hemorrhagic stroke							
<90	17/701 (2.4)	19/938 (2.0)	0.88 (0.45–1.72)	0.715	0.93 (0.41–2.11)	0.863	0.831
90–110	12/393 (3.1)	11/751 (1.5)	0.48 (0.21–1.08)	0.076	0.47 (0.21–1.28)	0.152	
≥110	1/69 (1.4)	3/124 (2.4)	1.67 (0.17–16.02)	0.658	13.28 (0–Inf)	1.0	
All bleeding							
<90	49/701 (7.0)	75/938 (7.9)	1.16 (0.81–1.67)	0.415	1.13 (0.74–1.71)	0.569	0.565
90–110	28/393 (7.1)	64/751 (8.5)	1.20 (0.77–1.87)	0.428	1.15 (0.70–1.90)	0.575	
≥110	3/69 (4.3)	8/124 (5.6)	1.34 (0.40–5.19)	0.670	0.78(0.11–5.47)	0.804	

### BP variability

**Fig. 3:**
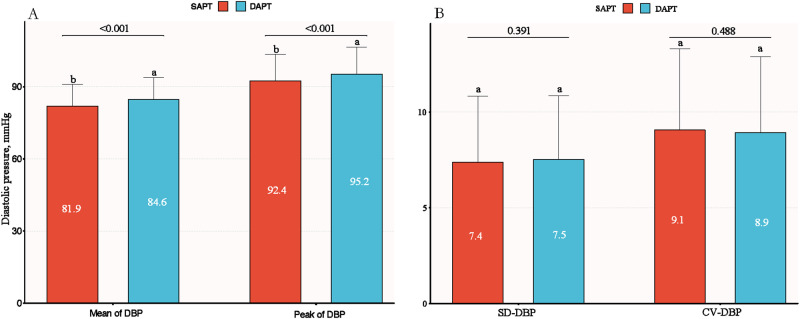
Comparison of diastolic blood pressure variability between treatment groups within 3 days of admission. **A** indicate the comparison mean of DBP and peak of DBP; **B** indicate the comparison SD and CV of DBP. *SD standard deviation, CV coefficient of variation

The variation in DBP within 3 days of admission between different treatment groups is shown in Fig. 3. The mean and peak diastolic BP within 3 days were statistically significant in two treatment groups (*P* values < 0.001). However, there was no significant difference in diastolic BP variation SD or CV within 3 days between the two treatment groups, which were 7.4 mmHg and 9.1% in the SAPT group and 7.5 mmHg and 8.9% in the DAPT group (*P* values > 0.05). Systolic BP showed similar results between the two treatments (Supplementary Fig. 3). The patients had a higher level of systolic BP variability of SD (10.3 ± 4.7 mmHg vs 11.4 ± 5.6 mmHg, *P* = 0.009) and CV (7.1 ± 3.2% vs 7.7 ± 3.7%, *P* = 0.04) when they had a composite vascular event during follow-up, but not in diastolic BP variability of SD (Supplementary Table 6).

## Discussion

In this study, we found that patients with DBP between 90 and 110 mmHg benefit more from DAPT regarding composite vascular events than patients with DBP < 90 mmHg or DBP ≥ 110 mmHg. DAPT did not increase the risk of bleeding in patients with higher BP levels on admission.

Our findings were inconsistent with previous studies. The CHANCE and POINT trial subgroups were also investigated for baseline BP and DAPT treatment. In the CHANCE subgroup, patients with SBP greater than 140 mmHg or DBP greater than 90 mmHg after minor stroke or TIA benefitted more from DAPT [8]. The analysis did not report a formal interaction term for SBP or DBP treatment. In contrast, the POINT subgroup showed a more significant benefit of DAPT in patients with SBP less than 140 mmHg but not in those with a DBP less than 90 mmHg, and the interaction between baseline BP and treatment was significant [9]. The contradictory conclusions may be due to the varied incidence of intracranial stenosis among different races. The CHANCE trial mainly enrolled Asian populations, who have a higher proportion of intracranial arterial stenosis [13], which requires higher BP to maintain intracranial perfusion pressure after ischemic stroke [13], especially for patients with multiple stenoses of cerebral arterie [1, 14]. However, there was no significant difference in the response to the two antiplatelet therapies between patients with and without ICAS in the CHANCE trial [15], which indicates that the effect of BP and ICAS on stroke outcomes should be considered together. Studies have shown that low BP levels (SBP/DBP < 140/90 mm Hg) are harmful in early AIS with hypertension and carotid stenosis (CAS), especially in patients with CAS ≥ 50% [16]. In our study, the proportion of ICAS in patients with different diastolic BP levels was not significantly different. Although there was a significant difference in the rate of intracranial artery stenosis between SBP groups, it did not interact with treatment regimens. As a result, the presence of ICAS is not the only indicator that affects the relationship between BP and stroke prognosis in our study, even though previous studies have suggested that the hypertensive response seen in patients is essential for maintaining cerebral perfusion pressure. This may be the reason why the control of cerebral perfusion is complex and involves a myriad of interwoven mechanisms [17].

Why does BP level affect the efficacy of DAPT in patients with minor stroke? The exact underlying mechanism remains unclear. In some trials, lowering BP has been shown to be beneficial in the treatment of acute strokes (target SBP less than 140 mmHg or less than 160 mmHg) [18]. The combination of DAPT’s antithrombotic properties may reduce the risk of early stroke recurrence by reducing embolic events and stabilizing the endothelium simultaneously [19]. Others have assumed that the protective mechanism of poststroke stress hypertension may amplify the efficacy of DAPT, and a higher BP may improve collateral circulation distal to an arterial occlusion and, as a result, prevent adverse outcomes [20, 21]. At the same time, animal experiments have found that the activation of platelets is affected by both low blood flow and high blood flow, and there are many microRNAs on the surface of platelets [22]. The abnormal expression of certain microRNAs is related to both platelet reactivity [23, 24] and BP regulation [25, 26]. These physiological mechanisms may explain part of the phenomenon. There is a complex relationship between DBP and stroke outcomes according to our study [27], which might partly explain the variable results from previous studies, but should inform future studies as well.

Our study found that DBP but not SBP interacted with dual antiplatelet therapy. A possible reason is that our study did not set an age limit, whereas the CHANCE and POINT studies included patients 40 years or older [9, 13]. In our study, patients with DBP in the 90–110 mmHg range were younger than those with DBP less than 90 mmHg (59.9 ± 11.0 vs. 63.8 ± 12.1, *P* < 0.001). DBP declines naturally after the age of 55, and pulse pressure rises as a result [27]. A possible explanation for our finding is that a younger age, represented by moderately elevated DBP, may have a reduced level of residual on-treatment platelet reactivity [28]. This post-hoc analysis included only hypertensive subjects over 50 years of age in SPRINT, showed a U-shaped relationship between baseline DBP and the primary composite outcome [7]. We further analyzed the interaction effect between age, diastolic pressure level and antiplatelet therapy, and revealed that only patients aged more than 55, with DBP in the 90–110 mmHg range, can benefit from DAPT (Supplementary Fig. 2), which is consistent with the SPRINT findings. To further validate our results, we analyzed data on BP variability within 72 hours of admission. The results showed that the BP variability between the different treatment groups was not significantly different. The POINT and CHANCE subgroups did not provide this partial result. However, these indicators all depend on the mean arterial pressure, so further analysis of some BP variation indices independent of the mean can be performed in the future. In this population, DAPT treatment did not increase the risk of severe bleeding events. However, the rate of hemorrhagic stroke was higher in the SAPT group than in the DAPT group, which was different from the results of previous randomized controlled trials. It may be that in the real world, DAPT is widely used, but people at high bleeding risk are usually not given DAPT [29]. At the same time, real-world studies have found that not all patients in DAPT group use load dose of 300 mg clopidogrel [30], which may also reduce the risk of bleeding to a certain extent.

### Perspective of Asia

The relationship between BP and stroke is complex, impacting both prognosis [31, 32] and responsiveness to antiplatelet therapy [8, 9]. This complexity may explain the inconsistent findings in trials of antihypertensive treatment during the acute phase of ischemic stroke [33]. Research suggests that specific platelet-driven biomarkers may interact with acute BP levels, contributing to stroke outcomes [22, 23]. Our study revealed that maintaining mild-moderate diastolic BP levels is more advantageous for patients with mild stroke from DAPT. This groundbreaking finding might provide a new perspective for clinical decision-making and personalized stroke treatment. Although no significant results were observed in the systolic pressure group, our findings are consistent with supplementary analysis from the POINT trial [9]. Additionally, the ENCHANTED2/MT experiment from China found that optimal BP levels during the acute phase after endovascular thrombectomy for AIS may range from 140–180mmHg [34]. These findings highlight the importance of considering baseline BP levels when deciding on dual antiplatelet therapy and suggest the need for further exploration of potential mechanisms.

The efficacy of DAPT in reducing stroke recurrence may vary in patients with acute mild stroke and different BP levels. Previous studies have reported varying associations between Asian and European populations [8, 9]. Therefore, more research is necessary to confirm the positive impact of mild-moderate diastolic BP levels on stroke recurrence reduction in other populations.

This cohort study has some limitations. Data analysis in this study was not designed to answer the proposed question, which introduces inherent biases. This may produce an unexpected result. For a more comprehensive evaluation of the observed association, we also had additional BP measurements collected 3 days after stroke onset. At the same time, we performed a sensitivity analysis after matching the propensity score of the data, and the results were consistent. Another limitation is that the analysis is based on the ITT principle, and patients may change the antiplatelet treatment plan during follow-up, which may bias the results.

## Conclusion

In this cohort study based on the real world, patients with a DBP greater than 90 mmHg and less than or equal to 110 mmHg were more beneficial from DAPT than to those with lower baseline DBPs, particularly in reducing risk of ischemic stroke recurrences at 90 days. The generalizability of our findings needs to be confirmed through independent studies.

### Supplementary information

Supplemental Material

Supplementary Appendix Study Protocol

STROBE Statement
