# Supplementary material for: Minor stroke patients with mild-moderate diastolic blood pressure derive greater benefit from dual antiplatelet therapy
Source: Hypertens Res. 2023 Sep 5;47(2):291–301. doi: 10.1038/s41440-023-01422-8 (PMC10838769; doi:10.1038/s41440-023-01422-8)

Supplemental Material: Minor Stroke Patients with a Mild-Moderate Diastolic Blood Pressure Level Derive Greater Benefit from Dual Antiplatelet Therapy

List of Supplemental Tables and Figures

Supplement Table 1. Baseline Characteristic of Patients Stratified by Antiplanet Therapy

Supplement Table 2. Baseline Characteristic of Patients Stratified by Systolic Blood Pressure

Supplement Table 3. Baseline Characteristic of Patients Stratified by Antiplanet Therapy after PSM

Supplement Table 4. Efficacy Outcomes of Patients with Different Antiplatelet Therapies Stratified by baseline SBP and the Model with the Interaction Term of SBP and treatment

Supplement Table 5. Safety Outcomes of Patients with Different Antiplatelet Therapies Stratified by baseline SBP and the Model with the Interaction Term of SBP and treatment

Supplement Table 6. Characteristic of Patients Stratified by whether have composite vascular events during follow-up.

Supplement Figure 1 Hazard Ratio for the Primary Outcome in Subgroups

Supplement Figure 2 Hazard Ratio for the Primary Outcome in Age × DBP Subgroups

Supplement Figure 3 Comparison of SBP variability between treatment groups within 3 days of admission

Supplement Figure 4 COX model for primary outcome after PSM, stratified DBP and SBP with events rates for SAPT and DAPT

Supplement Table 1. Baseline Characteristic of Patients Stratified by Antiplanet Therapy

Variables	Total	SAPT	DAPT	P value

	(n = 2976)	(n = 1163)	(n = 1813)	
Age, years	61.7 ± 11.9	64.9 ± 12.7	59.7 ± 11.0	<0.001
Sex, n (%)				<0.001
Female	794 (26.6)	355 (30.5)	439 (24.2)	
Male	2182 (73.3)	808 (69.5)	1374 (75.8)	
BMI, kg/m^2	24.9 ± 3.6	24.5 ± 3.6	25.1 ± 3.5	<0.001
Systolic pressure, mmHg	152.8 ± 21.8	152.0 ± 22.3	153.4 ± 21.6	0.083
Diastolic pressure, mmHg	88.4 ± 13.7	87.0 ± 13.6	89.3 ± 13.6	<0.001
Smoking status, no. (%)				<0.001
Never	1420 (48.0)	618 (53.5)	802 (44.5)	
Previous smoking	182 (6.2)	71 (6.1)	111 (6.2)	
Current smoking	1285 (43.4)	434 (37.5)	853 (47.3)	
**Medical history, no. (%)**				
Hypertension	1803 (60.6)	726 (62.4)	1077 (59.4)	0.110
Diabetes mellitus	791 (26.6)	325 (27.9)	466 (25.7)	0.177
Lipid disorder	69 (2.3)	24 (2.1)	45 (2.5)	0.541
AF	16 (0.5)	11 (0.9)	5 (0.3)	0.015
TIA	49 (1.6)	17 (1.5)	32 (1.8)	0.599
IS	676 (22.7)	311 (26.7)	365 (20.1)	<0.001
PAD	24 (0.8)	11 (0.9)	13 (0.7)	0.609
AM	48 (1.6)	22 (1.9)	26 (1.4)	0.334

CAD	164 (5.5)	73 (6.3)	91 (5.1)	0.142
ICH	64 (2.2)	42 (3.6)	22 (1.2)	<0.001
**Medication history use, no. (%)**				
Antiplanet	335 (11.3)	141 (12.1)	194 (10.7)	0.307
Anticoagulated	3 (0.1)	3 (0.3)	0 (0)	0.073
Antihypertensive	1229 (41.3)	499 (42.9)	730 (40.2)	0.159
Statins	223 (7.5)	98 (8.4)	125 (6.9)	0.165
**Clinical evaluation, no. (%)**				
Baseline NIHSS score				0.537
≤ 3	2411 (81.0)	948 (81.5)	1463 (80.7)	
4–5	565 (19.0)	215 (18.5)	350 (19.3)	
Onset to arrival time				<0.001
≤24 h	1670 (56.1)	593 (51.0)	1077 (59.4)	
24–72 h	1306(43.8)	570 (49.0)	736 (40.6)	
Pre-stroke mRS				0.060
0	2533 (85.1)	968 (83.2)	1565 (86.3)	
1	368 (12.4)	161 (13.8)	207 (11.4)	
2	75 (2.5)	34 (2.9)	41 (2.3)	
TOAST				0.002
LAA	998 (30.2)	373 (32.1)	525 (29.0)	
SVO	1368 (46.0)	485 (41.7)	883 (48.7)	

OE/UD	709 (23.8)	305 (26.2)	404 (22.2)	
ICAS				0.001
No	1688 (56.7)	615 (52.8)	1073 (59.2)	
Yes	1182 (39.7)	496 (42.6)	686 (37.8)	
**Laboratory results (Mean ± SD)**				
LDL-C, mmol/L	2.6 ± 0.8	2.6 ± 0.9	2.6 ± 0.8	0.713
HCY, µmol/L	24.0 ± 21.4	23.7± 21.9	24.1 ± 21.0	0.606
Creatinine, µmol/L	74.7 ± 36.6	76.7 ± 44.3	73.4 ± 30.7	0.029
Urea, mmol/L	5.6 ± 11.9	5.5 ± 2.0	5.7 ± 15.1	0.645
INR	1.1 ± 0.4	1.1 ± 0.2	1.1 ± 0.4	0.56
WBC, 10^9/L	7.1 ± 2.2	7.1 ± 2.3	7.2 ± 2.2	0.925
PLT, 10^9/L	217.2 ± 65.4	214.5 ± 69.0	218.9 ± 62.9	0.087

Supplement Table 2. Baseline Characteristic of Patients Stratified by Systolic Blood Pressure

Variables	< 140 mmHg (n = 860)	140–180 mmHg (n = 1774)	≥180 mmHg (n = 346)	P value
Age, years	60.9 ± 12.0	62.0 ± 11.7	62.5 ± 12.8	0.044
Sex, n (%)				0.188
Female	214 (24.9)	477 (26.9)	103 (29.8)	
Male	646 (75.1)	1297 (73.1)	243 (70.2)	
BMI, kg/m^2	24.3 ± 3.5	25.1 ± 3.5	25.2 ± 3.9	< 0.001

Systolic pressure, mmHg	128.0 ± 9.3	157.4 ± 10.9	191.9 ± 11.1	< 0.001
Diastolic pressure, mmHg	78.8 ± 9.4	90.5 ± 12.2	101.2 ± 14.3	< 0.001
Smoking, no (%)				0.028
Never	369 (43.1)	872 (49.5)	180 (52.6)	
Previous smoking	63 (7.4)	104 (5.9)	16 (4.7)	
Current smoking	404 (47.1)	744 (42.2)	139 (40.6)	
**Medical history, n (%)**				
Hypertension	417 (48.5)	1130 (63.7)	258 (74.5)	< 0.001
Diabetes mellitus	203 (23.6)	488 (27.5)	101 (29.2)	0.049
Lipid disorder	21 (2.4)	39 (2.2)	9 (2.6)	0.852
AF	3 (0.3)	10 (0.6)	3 (0.9)	0.74
TIA	21 (2.4)	25 (1.4)	3 (0.9)	0.071
IS	213 (24.8)	401 (22.6)	62 (17.9)	0.036
PAD	8 (0.9)	15 (0.8)	1 (0.3)	0.570
AM	15 (1.7)	30 (1.7)	5 (1.4)	0.933
CAD	54 (6.3)	98 (5.5)	13 (3.8)	0.243
ICH	20 (2.3)	35 (2.0)	9 (2.6)	0.68
**Medication use, n (%)**				
Antiplanet	107 (12.4)	201 (11.3)	29 (8.4)	0.095
Anticoagulated	0 (0)	3 (0.2)	0 (0)	0.104
Antihypertensive	297 (34.5)	772 (43.5)	160 (46.2)	< 0.001

Statins	76 (8.8)	131 (7.4)	17 (4.9)	0.150
**Medication onset, n (%)**				
Antiplatelet				0.014
SAPT	371 (43.1)	661 (37.3)	132 (38.2)	
DAPT	489 (56.9)	1113 (62.7)	214 (61.8)	
Statin	855 (99.4)	1762 (99.3)	342 (98.8)	0.503
Duration of DAPT, days	36.8 ± 33.2	32.8 ± 31.7	34.6 ± 32.5	0.068
**Clinical evaluation, n (%)**				
Baseline NIHSS score				0.004
≤3	717 (83.4)	1438 (81.1)	260 (75.1)	
4–5	143 (16.6)	336 (18.9)	86 (24.9)	
Onset to arrival time				0.01
≤24 h	466 (54.2)	988 (55.7)	220 (63.6)	
24–72 h	394 (45.8)	786 (44.3)	126 (36.4)	
Pre-stroke mRS				0.654
0	734 (85.3)	1501 (84.6)	301 (87.2)	
1	102 (11.9)	229 (12.9)	38 (11.0)	
2	24 (2.8)	44 (2.5)	6 (1.7)	
TOAST				0.051
LAA	272 (31.6)	511 (28.8)	117 (33.8)	
SVO	370 (43.0)	855 (48.2)	145 (41.9)	

OE/UD	218 (25.3)	407 (23.0)	84 (24.3)	
ICAS				< 0.001
No	524 (60.9)	1002 (56.5)	164 (47.4)	
Yes	306 (35.6)	715 (40.3)	163 (47.1)	
**Laboratory results (Mean ± SD)**				
LDL-C, mmol/L	2.5 ± 0.8	2.6 ± 0.8	2.7 ± 0.9	< 0.001
HCY, µmol/L	24.6 ± 23.4	23.9 ± 20.9	22.5 ± 18.0	0.328
Creatinine, µmol/L	73.7 ± 34.7	75.1 ± 39.0	75.7 ± 27.9	0.612
Urea, mmol/L	6.2 ± 21.7	5.3 ± 2.1	5.4 ± 2.1	0.287
INR	1.1 ± 0.2	1.1 ± 0.4	1.1 ± 0.3	0.216
WBC, 10^9/L	7.1 ± 2.5	7.1 ± 2.1	7.5 ± 2.2	0.036
PLT, 10^9/L	219.4 ± 68.9	216.1 ± 63.6	217.5 ± 65.2	0.513

Supplement Table 3 Baseline Characteristic of Patients Stratified by Antiplatelet Therapy after PSM

Variables	Total (n = 1724)	SAPT (n = 862)	DAPT (n = 862)	P value
Age, years	62.9 ± 11.2	62.6 ± 11.9	63.3 ± 10.4	0.201
Sex, n (%)				0.710
Female	499 (28.9)	246 (28.5)	253 (29.4)	
Male	1225 (71.1)	616 (71.5)	609 (70.6)	

BMI, kg/m2	24.8 ± 3.5	24.8 ± 3.6	24.9 ± 3.4	0.582
Systolic pressure, mmHg	156.2 ± 22.3	151.9 ± 22.4	160.4 ± 21.4	< 0.001
Diastolic pressure, mmHg	91.5 ± 13.8	87.8 ± 13.5	95.2 ± 13.1	< 0.001
Smoking status, no. (%)				0.123
Never	902 (52.3)	429 (49.8)	473 (54.9)	
Previous smoking	104 (6.0)	56 (6.5)	48 (5.6)	
Current smoking	669 (38.8)	355 (41.2)	314 (36.4)	
Medical history, no. (%)				
Hypertension	1068 (61.9)	511 (59.3)	557 (64.6)	0.022
Diabetes mellitus	463 (26.9)	236 (27.4)	227 (26.3)	0.625
Lipid disorder	38 (2.2)	19 (2.2)	19 (2.2)	1.000
AF	5 (0.3)	1 (0.1)	4 (0.5)	0.374
TIA	30 (1.7)	17 (2)	13 (1.5)	0.461
IS	416 (24.1)	194 (22.5)	222 (25.8)	0.115
PAD	15 (0.9)	7 (0.8)	8 (0.9)	0.795
AM	24 (1.4)	12 (1.4)	12 (1.4)	1.000
CAD	99 (5.7)	49 (5.7)	50 (5.8)	0.918
ICH	43 (2.5)	23 (2.7)	20 (2.3)	0.643
**Medication history use, n (%)**				
Antiplanet	198 (11.5)	104 (12.1)	94 (10.9)	0.817
Anticoagulated	1 (0.1)	1 (0.1)	0 (0)	1.000

Antihypertensive	710 (41.2)	349 (40.5)	361 (41.9)	0.781
Statins	131 (7.6)	69 (8)	62 (7.2)	0.714
**Clinical evaluation, no. (%)**				
Baseline NIHSS score				0.951
≤3	1405 (81.5)	702 (81.4)	703 (81.6)	
4–5	319 (18.5)	160 (18.6)	159 (18.4)	
Onset to arrival time				<0.001
≤24 h	967 (56.1)	446 (51.7)	521 (60.4)	
24–72 h	757 (43.9)	416 (48.3)	341 (39.6)	
Pre-stroke mRS				0.072
0	1473 (85.4)	734 (85.2)	739 (85.7)	
1	214 (12.4)	110 (12.8)	104 (12.1)	
2	37 (2.1)	18 (2.1)	19 (2.2)	
TOAST				<0.001
LAA	505 (29.3)	273 (31.7)	232 (26.9)	
SVO	807 (46.8)	366 (42.5)	441 (51.2)	
OE/UD	412 (23.9)	223 (25.9)	189 (21.9)	
ICAS				0.200
No	959 (55.6)	462 (53.6)	497 (57.7)	
Yes	706 (41.0)	367 (42.6)	339 (39.3)	
**Laboratory results (Mean ± SD)**				

LDL-C, mmol/L	2.6 ± 0.8	2.6 ± 0.8	2.6 ± 0.8	0.741
HCY, µmol/L	23.6 ± 21.6	23.0 ± 20.1	24.2 ± 22.9	0.249
Creatinine, µmol/L	75.0 ± 33.8	74.4 ± 27.4	75.5 ± 39.1	0.508
Urea, mmol/L	5.3 ± 2.0	5.3 ± 1.8	5.3 ± 2.2	0.655
INR	1.1 ± 0.5	1.1 ± 0.5	1.1 ± 0.5	0.712
WBC, 109/L	7.0 ± 2.2	7.1 ± 2.3	7.0 ± 2.1	0.155
PLT, 109/L	216.7 ± 65.5	217.2 ± 69.1	216.1 ± 61.8	0.730

Propensity score matching (PSM) was performed with the following covariates: age, sex, BMI, baseline blood pressure, NIHSS, onset to arrival time, pre-stroke mRS, statin use, smoking status, HTN, DM, lipid disorder, AF, TIA, IS, PAD, AM, CAD, ICH, previous antiplatelet treatment, previous antihypertensive treatment, previous statin treatment, Crea, LDL.

4

Supplement Table 4 Efficacy Outcomes of Patients with Different Antiplatelet Therapies Stratified by baseline SBP and the Model with the Interaction Term of SBP and treatment

Outcome	SAPT event, no. (%)/total no.	DAPT event, no. (%)/total no.	Crude HR (95%CI)	Crude P value	Adj HR (95%CI)	Adj P value	P for interaction
SBP level, mmHg							
**Primary outcome**							
**Composite vascular events**							
<140	30/370 (8.1)	31/488 (6.4)	0.82 (0.49~1.35)	0.429	1.07 (0.61~1.87)	0.817	0.719
140-180	58/661 (8.8)	89/1112 (8.0)	0.89 (0.64~1.24)	0.483	0.99 (0.69~1.45)	0.970	

≥180	15/132 (11.4)	34/213 (16.0)	1.43 (0.78~2.62)	0.251	1.39 (0.69~2.81)	0.280	
**Secondary outcomes**							
All stroke							
<140	27/370 (7.3)	30/488 (6.1)	0.88 (0.52~1.49)	0.637	1.12 (0.62~2.01)	0.705	0.909
140-180	57/661 (8.6)	86/1112 (7.7)	0.87 (0.62~1.22)	0.434	0.98 (0.68~1.43)	0.937	
≥180	15/132 (11.4)	32/213 (15.0)	1.34 (0.73~2.48)	0.349	1.26 (0.62~2.54)	0.415	
Ischemic stroke							
<140	26/370 (7.0)	27/488 (5.5)	0.82 (0.48~1.42)	0.480	1.01 (0.55~1.85)	0.967	0.870
140-180	48/661 (7.3)	80/1112 (7.2)	0.96 (0.67~1.38)	0.828	1.07 (0.72~1.60)	0.727	
≥180	14/132 (10.6)	30/213 (14.1)	1.34 (0.71~2.53)	0.361	1.37 (0.66~2.83)	0.311	
TIA							
<140	1/370 (0.3)	3/488 (0.6)	2.28 (0.24~21.95)	0.475	5.37 (0.27~97.43)	0.284	0.058
140-180	7/661 (1.1)	4/1112 (0.4)	0.34 (0.1~1.16)	0.084	0.28 (0.06~1.49)	0.173	
≥180	0/132 (0)	2/213 (0.9)	Inf (0~Inf)	0.999	62.02 (0~Inf)	1	
Symptomatic intracerebral hemorrhage							

<140	0/370 (0)	0/480 (0)	1 (1~1)	-	1 (1~1)	-	0.249
140-180	2/661 (0.3)	2/1112 (0.2)	1.19 (0.11~13.11)	0.888	2.05 (0.16~ 25.47)	0.370	
≥180	1/132 (0.8)	0/213 (0)	0 (0~Inf)	0.999	0 (0~Inf)	1	
Myocardial infarction or angina attacks							
<140	1/370 (0.3)	1/488 (0.2)	0.76 (0.05~12.11)	0.844	0.75 (0.02~ 26.54)	0.843	0.490
140-180	1/661 (0.2)	2/1112 (0.2)	1.19 (0.11~13.11)	0.888	1.1 (0.08~ 15.63)	0.912	
≥180	0/132 (0)	2/213 (0.9)	Inf (0~Inf)	0.999	3.64 (0~ Inf)	0.970	
Vascular death							
<140	2/370 (0.5)	0/488 (0)	0 (0~Inf)	0.999	0 (0~Inf)	1	0.008
140-180	0/661 (0)	1/1112 (0.1)	Inf (0~Inf)	0.999	1 (1~1)	1	
≥180	0/132 (0)	0/213 (0)	1 (1~1)	-	1 (1~1)	-	

*Adjusted for factors of sex, age, BMI, diastolic blood pressure, baseline NIHSS, onset time to hospital arrival, statin use at admission, smoking, previous IS, ICH, AF, creatinine, ICAS, TOAST.

Supplement Table 5 Safety Outcomes of Patients with Different Antiplatelet Therapies Stratified by baseline SBP and the Model with the Interaction Term of SBP and treatment

Outcome	SAPT event, no. (%)/total no.	DAPT event, no. (%)/total no.	Crude HR (95%CI)	Crude P value	Adj HR (95%CI)	Adj P value	P for interaction
SBP level, mmHg							

**Safety outcomes**							
Sever bleeding							
<140	0/370 (0)	0/488 (0)	1 (1~1)	-	1 (1~1)	-	0.520
140-180	2/661 (0.3)	2/1112 (0.2)	1.19 (0.11~13.11)	0.888	2.05 (0.24~25.47)	0.370	
≥180	1/132 (0.8)	0/213 (0)	0 (0~Inf)	0.999	0(0~Inf)	1	
Hemorrhagic stroke							
<140	8/370 (2.2)	10/488 (2.0)	0.95 (0.37~2.40)	0.906	1.01 (0.31~3.11)	0.994	0.580
140-180	19/661 (2.9)	18/1112 (1.6)	0.59 (0.31~1.13)	0.113	0.57 (0.27~1.23)	0.154	
≥180	3/132 (2.3)	5/213 (2.3)	1.03 (0.25~4.31)	0.967	0.68 (0.06-9.53)	0.772	
All bleeding							
<140	27/370 (7.3)	46/488 (9.4)	1.29 (0.80~2.07)	0.299	1.39 (0.80~2.41)	0.238	0.180
140-180	47/661 (7.1)	81/1112 (7.3)	1.04 (0.73~1.50)	0.822	0.89 (0.59~1.34)	0.574	
≥180	6/132 (4.5)	20/213 (9.3)	2.01 (0.85~5.26)	0.108	2.62 (0.82~8.4)	0.105	

7

Supplement Table 6 Characteristic of Patients Stratified by whether have composite vascular events during

follow-up				
Variables	Total (n = 2976)	No composite vascular events during follow-up (n = 2719)	Composite vascular events during follow-up (n = 257)	P value
Age, years	61.7 ± 11.9	61.6 ± 11.9	62.7 ± 11.9	0.174
Sex, n (%)				0.603
Female	794 (26.7)	722 (26.6)	72 (28)	
Male	2182 (73.3)	1997 (73.4)	185 (72)	
BMI, kg/m2	24.8 ± 3.5	24.8 ± 3.6	24.9 ± 3.4	0.582
**Blood pressure variables**				
Baseline SBP, mmHg	156.2 ± 22.3	151.9 ± 22.4	160.4 ± 21.4	< 0.001
Mean SBP	145.1 ± 14.1	144.8 ± 14.0	147.8 ± 14.8	0.021
Peak SBP	160.2 ±17.3	159.9 ± 17.1	164.5 ± 19.5	0.004
SD SBP	10.4 ± 4.8	10.3 ± 4.7	11.4 ± 5.6	0.009
CV SBP	7.1 ± 3.2	7.1 ± 3.2	7.7 ± 3.7	0.04
Baseline DBP, mmHg	91.5 ± 13.8	87.8 ± 13.5	95.2 ± 13.1	< 0.001
Mean DBP	83.6 ± 9.1	83.6 ± 9.0	84.2 ± 10.0	0.46
Peak DBP	94.4 ± 11.0	94.3 ± 11.0	94.8 ± 11.5	0.655
SD DBP	7.5 ± 3.3	7.5 ± 3.3	7.5 ± 3.4	0.781
CV DBP	9.1 ± 4.0	9.1 ± 4.0	8.9 ± 4.0	0.654
Smoking status, no. (%)				0.065

Never	1420 (48.0)	1301 (48.1)	119 (46.9)	
Previous smoking	182 (6.2)	159 (5.9)	23 (9.1)	
Current smoking	1285 (43.4)	1183 (43.8)	102 (40.2)	
Medical history, no. (%)				
Hypertension	1803 (60.6)	1638 (60.2)	165 (64.2)	0.213
Diabetes mellitus	791 (26.6)	693 (25.5)	98 (38.1)	< 0.001
Lipid disorder	69 (2.3)	58 (2.1)	11 (4.3)	0.028
AF	16 (0.5)	12 (0.4)	4 (1.6)	0.043
TIA	49 (1.6)	43 (1.6)	6 (2.3)	0.310
IS	676 (22.7)	603 (22.1)	73 (28.4)	0.022
PAD	24 (0.8)	22 (0.8)	2 (0.8)	1.000
AM	48 (1.6)	45 (1.7)	3 (1.2)	0.797
CAD	164 (5.5)	151 (5.6)	13 (5.1)	0.726
ICH	64 (2.2)	60 (2.2)	4 (1.6)	0.494
**Medication history use, n (%)**				
Antiplanet	335 (11.3)	305 (11.2)	30 (11.7)	0.279
Anticoagulated	3 (0.1)	3 (0.1)	0 (0)	1.000
Antihypertensive	1229 (41.3)	1116 (41)	113 (44)	0.368
Statins	223 (7.5)	205 (7.3)	18 (7)	0.647
**Admission medication, no. (%)**				
Antiplatelet				

SAPT	1164 (39.1)	1060 (39)	103 (40.1)	0.727
DAPT	1813 (60.9)	1659 (61)	154 (59.9)	
Statin	2955 (99.3)	2700 (99.3)	255 (99.2)	0.702
**Clinical evaluation, no. (%)**				
Baseline NIHSS score				0.011
≤3	2411 (81.0)	2218 (81.6)	193 (75.1)	
4–5	565 (19.0)	501 (18.4)	64 (24.9)	
Onset to arrival time				0.038
≤24 h	1670 (56.2)	1510 (55.5)	160 (62.3)	
24–72 h	1306 (43.8)	1209 (44.5)	97 (37.7)	
Pre-stroke mRS				0.129
0	2533 (85.1)	2321 (85.4)	212 (82.5)	
1	369 (12.4)	327 (12)	41 (16)	
2	74 (2.5)	70 (2.6)	4 (1.6)	
TOAST				<0.001
LAA	898 (30.2)	793 (29.2)	105 (40.9)	
SVO	1368 (46.0)	1286 (47.3)	82 (31.9)	
OE/UD	709 (23.8)	639 (23.5)	70 (27.2)	
ICAS				< 0.001
No	1688 (56.7)	1583 (58.2)	105 (40.9)	
Yes	1182 (39.7)	1036 (38.1)	146 (56.8)	

**Laboratory results (Mean ± SD)**				
LDL-C, mmol/L	2.6 ± 0.8	2.6 ± 0.8	2.7 ± 0.8	0.116
HCY, µmol/L	23.9 ± 21.5	24.0 ± 21.5	23.0 ± 20.5	0.49
Creatinine, µmol/L	74.7 ± 36.2	74.6 ± 36.8	75.0 ± 29.9	0.874
Urea, mmol/L	5.6 ± 11.7	5.6 ± 12.3	5.4 ± 2.1	0.817
INR	1.1 ± 0.4	1.1 ± 0.5	1.1 ± 0.1	0.895
WBC, 10^9/L	7.1 ± 2.2	7.1 ± 2.2	7.5 ± 2.4	0.023
PLT, 10^9/L	217.4 ± 65.1	217.0 ± 65.2	221.8 ± 64.2	0.267

Supplement Figure 1 Hazard Ratio for the Primary Outcome in Subgroup

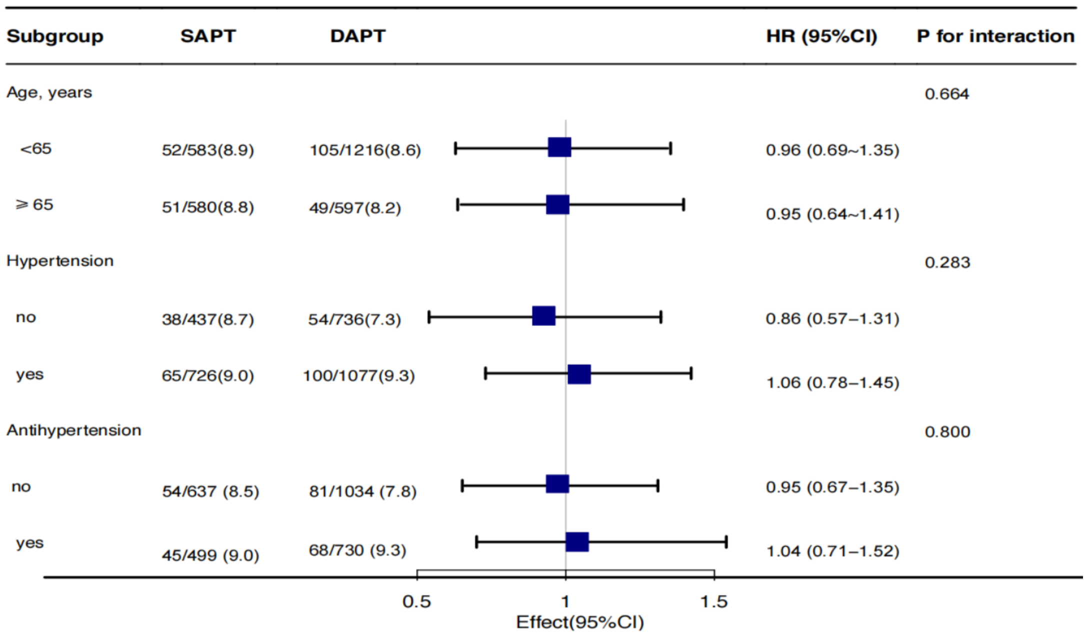

Supplement Figure 2 Hazard Ratio for the Primary Outcome in Age × DBP Subgroups

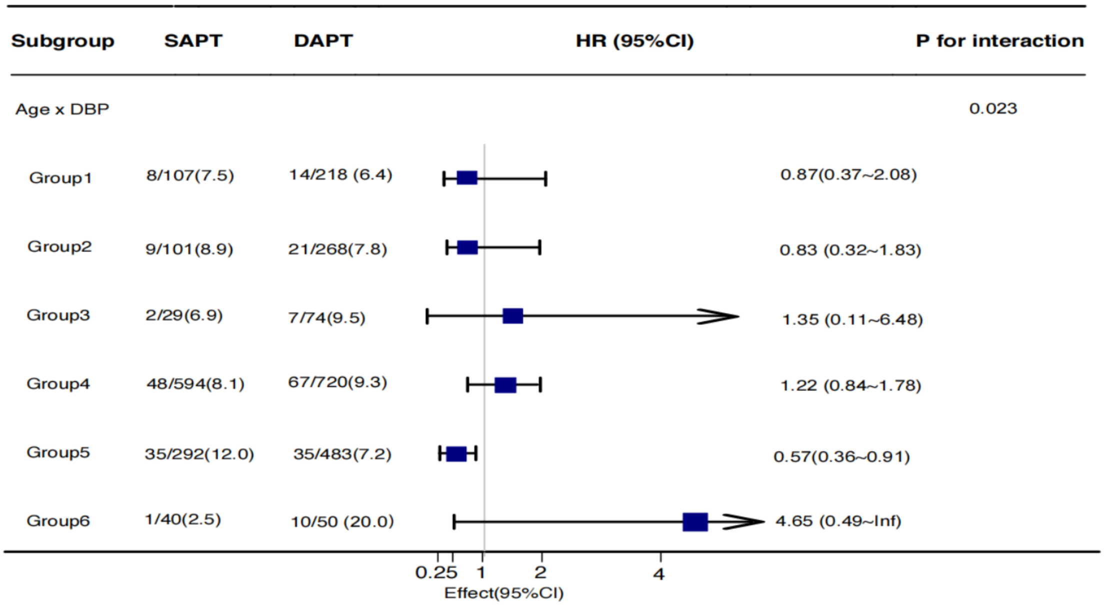

Group1 is patients younger than 55 years old and baseline DBP level less than 90mmHg; Group2 is patients younger than 55 years old and baseline DBP level between 90 to 110mmHg; Group3 is patients younger than 55 years old and baseline DBP equal to or more than 110 mmHg; Group4 is patients equal to or older than 55 years old and baseline DBP level less than 90mmHg; Group5 is patients equal to or older than 55 years old and DBP level between 90 to110mmHg; Group6 is patients equal to or older than 55 years old and DBP equal to or more than 110mmHg.

Supplement Figure 3 Comparison of SBP variability between treatment groups within 3 days of admission.

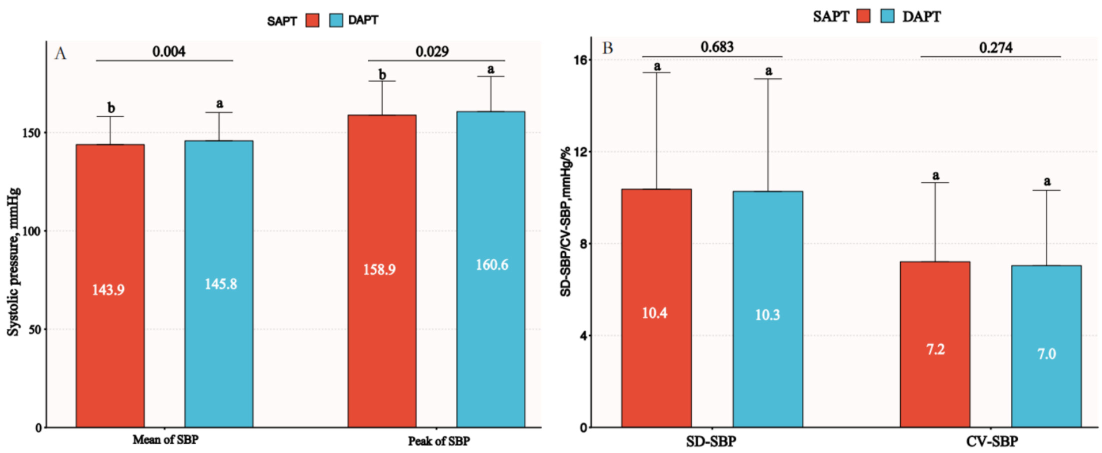

Figure A indicate the comparison mean of SBP and peak of SBP; Figure B indicate the comparison SD and CV of SBP. *SD, standard deviation; CV, coefficient of variation.

Supplement Figure 4 COX model for primary outcome after PSM, stratified DBP and SBP with events rates for SAPT and DAPT

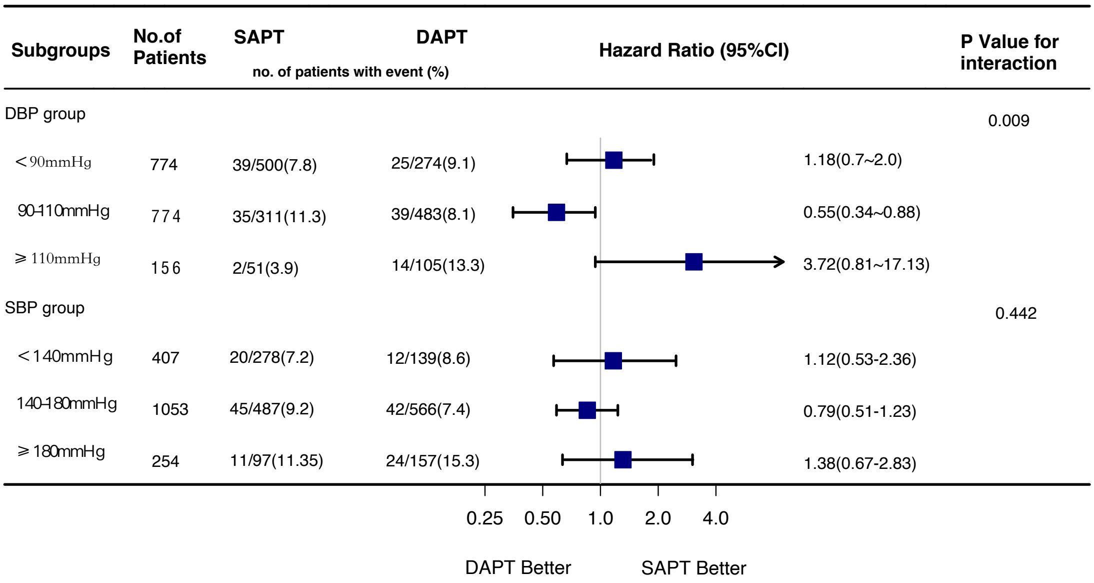

Supplement: Supplementary file 1 — Supplemental Material [file 41440_2023_1422_MOESM1_ESM.pdf]
