# Supplementary material for: Minor stroke patients with mild-moderate diastolic blood pressure derive greater benefit from dual antiplatelet therapy
Source: Hypertens Res. 2023 Sep 5;47(2):291–301. doi: 10.1038/s41440-023-01422-8 (PMC10838769; doi:10.1038/s41440-023-01422-8)
Supplement: Supplementary file 2 — Supplementary Appendix Study Protocol [file 41440_2023_1422_MOESM2_ESM.pdf]

Supplementary Appendix Study Protocol: Safety and efficacy of aspirin-clopidogrel in acute non-cardiogenic minor ischemic stroke: a prospective and multicenter study based on real-world (SEACOAST)

Introduction and rationale

Although cerebrovascular disease is the second most common cause of death in the world[1], it is the leading cause of death inChina[2]. Stroke has become a major public health problem that seriously impacts the national economy and people's livelihood in China because of its high incidence and recurrence rate and high rate of disability, complications, and mortality[3]. Presently, the diagnostic criteria of minor ischemic stroke (MIS) lacks consensus among researchers[4]. A NIHSS score ≤ 3 or ≤ 5 is considered to indicate MIS. The results of the 2012 CHANCE study showed that early dual antiplatelet therapy with aspirin plus clopidogrel within 24 hours of onset could effectively reduce the relative risk of recurrent stroke by 32% without an increased risk of hemorrhage for patients with MIS (NIHSS ≤ 3) or high-risk transient ischemic attack (ABCD2 > 4)[5]. The findings of the CHANCE study were confirmed in the POINT trial, and both enrolled patients with NIHSS scores ≤ 3 within 24 or 12 hours after onset[56] At present, intravenous thrombolysis therapy or intravascular mechanical thrombus therapy can lead to neurological function improvement, which is defined by a NIHSS score ≤ 5[7]. A NIHSS score of 5 indicates the beginning of disability[8]. However, whether thrombolysis is recommended for patients with a NIHSS score between 3 and 5 remains controversial, especially if deficits are not disabling. Early dual antiplatelet therapy may also be effective for secondary stroke prevention. However, if a stroke patient has a NIHSS score between 3 and 5 and an onset of 24 to 72 hours, the benefit of DAPT is unclear. This subgroup of stroke patients was not studied in the CHANCE or the POINT trial. Our questionnaire survey of neurologists showed that approximately 23% of patients with NIHSS scores ≤ 5 were treated with DAPT. Approximately 18% of neurologists would choose DAPT for patients with an onset within 24-72 hours. Approximately 52% of neurologists prescribed DAPT for patients with a NIHSS score between 3 to 5. Our single-center retrospective study of stroke patients with a NIHSS ≤ 5 and an onset time within 72 hours showed that 6.1% had recurrent strokes if treated with DAPT, compared to 10.8% in those who received aspirin only. Moreover, neurologists would

like to prescribe DAPT for stroke patients with a NIHSS score ≤ 5 and within 72 hours of onset. Therefore, there is a gap between the evidence found from two clinical trials and real-world clinical practice. The latest meta-analysis showed that stroke patients could benefit from receiving the benefits of DAPT could be used for stroke patients 3 days after onset[9], a time point at which deterioration of early neurological function would begin. A registry study in South Korea showed that more than 70% of patients (with 4≤ NIHSS ≤7) were treated with DAPT[10]. Last, a subgroup analysis of the CHANCE trial showed that the proportion of ICAS in China was as high as 55.8%[11], indicating that large artery stenosis is a cause of ischemic stroke in Chinese patients. Research on the benefit of DAPT for stroke patients with higher NIHSS scores and the expanded time window to begin treatment is ongoing[12]. However, the efficacy and safety of dual antiplatelet therapy for acute minor stroke (MIS patients with a NIHSS score ≤ 5 and onset within 72 hours) remain unclear.

Our study on the safety and efficacy of dual antiplatelet therapy in patients with acute noncardiogenic minor ischemic stroke (NIHSS score ≤ 5) (SEACOAST) is a multicenter study of the application of DAPT in real-world practice.

Methods

Design

SEACOAST is a multicenter, nonrandomized, and prospective registry trial in China intended to demonstrate the efficacy and safety of dual antiplatelet therapy for acute minor stroke (with NIHSS score ≤ 5 and onset within 72 hours). The main aim of the present study is to test the hypothesis that dual antiplatelet therapy should reduce the incidence of 90-day stroke recurrence and improve the functional outcomes of patients with acute minor stroke (MIS patients with a NIHSS score ≤ 5 and onset within 72 hours) but should not increase the risk of symptomatic intracranial hemorrhage.

All patients who meet the enrollment criteria will be invited to participate. The study was approved by the Ethics Committee of the First Hospital of Shanxi Medical University. Written informed consent will be signed prior to enrollment. Participants will be divided into two groups: the dual antiplatelet therapy (DAPT) group and the single antiplatelet therapy (SAPT) group. Patients with acute minor stroke (NIHSS score ≤ 5) and onset within 72 hours, as judged by the investigators, who received

antiplatelet therapy at approximately 5-10 stroke centers in China between October 2019 and November 2021 were enrolled in the trial. The detailed inclusion/exclusion criteria as follow. Study recruitment started in September 2019, and the estimated primary completion date was November 2021.

Ethics and dissemination

The patient or their guardian signed an informed consent form. The Ethics Committee of First Hospital of Shanxi Medical University approved the procedure, and approval was obtained from all other participating centers. The ethics approval number is 2019-SK004. The study protocol is registered at https://www.chictr.org.cn, Unique identifier: ChiCTR1900025214).

Study organization

The initial protocol was designed by the SEACOAST group and discussed by the academic team. The Steering Committee was composed of the Shanxi Stroke Association. Neuroimaging associated with clinical events was performed centrally and interpreted by three independents blinded neuroradiologists. The trial is partially supported by the Shanxi Stroke Association.

Patient population (Inclusion/Exclusion Criteria)

Inclusion Criteria:

1) acute minor ischemic stroke (NIHSS score ≤5 within 72 hours of symptom onset);
2) had been treated with antiplatelet drugs.
3) Informed consent signed.

(Symptom onset is defined by the "last seen normal" principle.)

Exclusion Criteria

1) Prestroke Modified Rankin Scale (mRs) Score > 2 (premorbid historical assessment);

2) Diagnosis of hemorrhage or other pathology, such as vascular malformation, tumor, abscess or other major nonischemic brain disease (e.g., multiple sclerosis) on baseline head CT or MRI.

3) TIA;

4) Clear indication for anticoagulation (presumed cardiac source of embolus, e.g.,

atrial fibrillation, prosthetic cardiac valves known or suspected endocarditis).

5) Patients who receive early intravenous thrombolysis or arterial thrombosis;

6) Currently or within 30 days before enrollment receiving an investigational drug or device.

7) No use or use of other antiplatelet drugs (except aspirin, clopidogrel);

8) Pregnant or lactating patients and patients planning to become pregnant within 90 days.

9) Those with severe mental disorders, unable to provide informed consent or unable to cooperate with the follow-up due to dementia.

10) Patients with complicated severe systemic disease with a life expectancy < 3 months.

11) Planned or likely revascularization (any angioplasty or vascular surgery) within 3 months.

Sample size determination

Our single-center study results indicated that the probability of stroke deterioration was approximately 10.8% in the aspirin group and 6.1% in the DAPT group. Considering the 10.8% recurrent stroke rate in the aspirin group and the 6.1% recurrent stroke rate in DAPT group, a p value < 0.05 indicating statistical significance at the 95% confidence interval, and a 10% drop out rate, the target number of participants will be at least 2500; PASS 22.0 will be used to perform sample size calculation.

Data collection

Data were collected retrospectively using the Research Electronic Data Capture (REDCap), which is a secure web-based software platform that supports data capture for research studies by providing (1) an intuitive interface for validated data capture, (2) audit trails for tracking data manipulation and export procedures, (3) automated export procedures for seamless data downloads to common statistical packages, and (4) procedures for data integration and interoperability with external sources. All researchers are trained in a unified manner. Data are currently registered in the web-based registry available at https://www.palacetrial.cn.

The following data were directly obtained from the registry database: (1)

demographics, including age, sex, body mass index, smoking, admission systolic blood pressure (SBP), and diastolic blood pressure (DBP); (2) medical history, including previous TIA, previous stroke, previous coronary artery disease (CAD), previous peripheral artery disease (PAD), hypertension (HTN), diabetes mellitus (DM), dyslipidemia, smoking, and atrial fibrillation (AF); (3) previous medication, including previous antiplatelet, anticoagulated, antihypertensive and statins medication use; (4) stroke characteristics, including the time from onset to arrival(categorized as ≤ 24 hours and among 24 hours to 72 hours), initial NIHSS scores (categorized as ≤ 3 score and between 4 to 5 score), prestroke mRS score, and ischemic stroke subtype according to the TOAST criteria; (5) laboratory data, including white blood cell counts, creatinine serum levels, platelet counts, international normalized ratio (INR), urea, homocysteine (Hcy), and fasting low-density lipoprotein cholesterol (LDL-C); (6) in-hospital treatment, including antiplatelet, lipid lowing, antidiabetic and antihypertensive therapy, and (7) in-hospital imaging evaluation, including cranial MRI/CT examination and vascular examination (TCD/MRA/CTA/DSA) helped to determine the presence of intracranial cerebral atherosclerosis (ICAS), determined by 50%–99% stenosis of large intracranial arteries according to Warfarin-Aspirin Symptomatic Intracranial Disease (WASID) trial criteria[13]; and (8) monitoring of some indicators, including daily blood pressure and blood glucose monitoring record results. For continuous variables, the missing value data were used by multiple imputation, and the optimal imputation data summarized after 5 interpolations were selected by the system for analysis.

Pharmaceutical regimen

The study subjects were divided into two groups for comparison according to the initial antiplatelet regimen: single antiplatelet therapy (SAPT) with monotherapy of aspirin (dose of 81mg/100mg/200mg/300mg) or clopidogrel (dose of 75mg/150mg/ 300mg) and dual antiplatelet therapy (DAPT) with aspirin (dose of 81mg/100mg/ 200mg/300mg) plus clopidogrel (load dose of 75mg/150mg/300mg).

Data management

Data quality control was divided into three parts: first, the patients' hospital information was retrieved from the electronic medical records system of each

subcenter; second, the trained researchers randomly performed cross data quality control every month; and third, three senior neurologists performed data quality control every month. We will use the data management system to manage the data. All data will be downloaded and analyzed at the First Affiliated Hospital of Shanxi Medical University.

Follow-up time

The follow-up time for the two groups will be 90 days and 1 year. Vascular events

were prospectively observed during hospitalization and during a 3-month follow-up period after the qualifying event via routine clinic visits or telephone interviews performed by experienced physicians with a predefined protocol. To ensure the accuracy of the outcome captured and to minimize inter-interviewer discrepancy, a set of uniform structured questionnaires was used by trained personnel. Enrollment will stop on November 31, 2021.

Outcomes

We set a composite vascular event (ischemic stroke, TIA, symptomatic intracerebral hemorrhage, myocardial infarction or angina attacks, and vascular death) as the primary outcome at 90 days. Recurrent ischemic stroke: (1) sudden onset of a new focal neurologic deficit, with clinical or imaging evidence of infarction lasting ≥24 hours and not attributable to a nonischemic cause (i.e., not associated with brain infection, trauma, tumor, seizure, severe metabolic disease, or degenerative neurologic disease), and (2) a new focal neurologic deficit lasting <24 hours and not attributable to a nonischemic cause but accompanied by neuroimaging evidence of new brain infarction. Imaging indicated that the new infarct should be geographically distinct from the original infarct[5]. Progressive ischemic stroke: rapid worsening of an existing focal neurologic deficit (NIHSS increasing ≥4, excluding hemorrhagic transformation after infarction or symptomatic intracranial hemorrhage) lasting >24 hours and not attributable to a nonischemic cause, accompanied by new ischemic changes from the initial infarct on baseline magnetic resonance imaging or computed

tomography of the brain[14]. Symptomatic intracerebral hemorrhage was defined as acute infiltration of blood into the brain parenchyma or subarachnoid space with associated neurological symptoms and imaging findings with an increase in the NIHSS score of four or more points[5]. Any subject who had rapid resolution of symptoms and no brain imaging suggesting tissue infarction was considered to have had a TIA. Any patient initially diagnosed with stroke who did not have further brain imaging with evidence of infarction but had complete resolution of symptoms within 24 hours were considered to have had a TIA[6]. Myocardial infarction was confirmed if one had more than two from below: typical chest pain, Troponin elevation, ECG changes (new ST segment changes, new Q wave, or new left bundle branch block)[15]. vascular death if definition as death due to vascular events[16]. Further efficacy exploratory analysis of mRS score changes (continuous) was performed and dichotomized at percentages of 0 to 2 versus 3 to 6 at the 3-month and one-year follow-ups.

Safety endpoints included severe bleeding incidence (Global Use of Strategies to Open Occluded Coronary Arteries definition), including severe bleeding and symptomatic intracranial hemorrhage; moderate bleeding (Global Use of Strategies to Open Occluded Coronary Arteries definition). Moderate hemorrhage was defined as bleeding that required transfusion of blood but did not lead to hemodynamic compromise requiring intervention[17]. Severe hemorrhage was defined as fatal or intracranial hemorrhage or other hemorrhage causing hemodynamic compromise that required blood or fluid replacement, inotropic support, or surgical intervention[18], and all bleeding events (intracerebral hemorrhage, skin bleeding, mucous membrane bleeding, gastrointestinal bleeding, and another visceral organ bleeding) during follow-up. All adverse events will be recorded.

Statistical analysis

Continuous variables that are normally distributed will be presented as the mean value and SD. When variables were not normally distributed, they were presented as medians and IQRs. Count data will be described with n (%). Student's t test was used for continuous variables, and the Pearson $\chi 2$ test was used for categorical variables. To compare the survival curve and survival rate of stroke recurrence, survival analysis will be conducted. Cox proportional hazard models will be used to explore the risk

factors for recurrent ischemic stroke. Propensity score matching will be used to control for confounding factors. All analyses were performed in SPSS 26.0 and the statistical software package R (http://www.R-project.org, The R Foundation).

Discussion

An important treatment strategy for patients with acute ischemic stroke is to prevent deterioration and recurrence of stroke. The treatment standard for patients with acute minor ischemic stroke has not been well established[19]. The CHANCE and POINT trials[56] have shown that DAPT is superior to aspirin alone in patients with minor ischemic stroke (NIHSS score ≤3) or high-risk TIA. A South Korean registration study showed that more than 70% of patients with a NIHSS score 4-7 treated with DAPT within 24 hours of onset were also affected[10]. Therefore, the optimal time frame to start DAPT post stroke to achieve efficacy remains unclear[420]. Patients with AIS can present within 24 hours, 48 hours, 72 hours, and even 7 days post onset[2122]. DAPT may be the only method for preventing early deterioration in late presenters[22] . A meta-analysis of dual versus mono antiplatelet therapy for acute non-cardioembolic IS or TIA patients within 3 days of symptom onset showed that compared with aspirin alone, DAPT was associated with a significant reduction in stroke recurrence (RR, 0.70; 95% CI, 0.59–0.82; P<0.001)[9]. The latest meta-analysis showed that DAPT can benefit patients if given within 3 days of onset.

In this proposed trial, loss to follow-up may occur. To prevent such a potential problem, the researchers will implement several methods for securing the follow-up. In addition, because of its multicenter design, confounding bias and selection bias will be well controlled. This trial to explore the efficacy and safety of dual antiplatelet therapy in patients with acute minor stroke (NIHSS score ≤ 5 and onset within 72 hours). Regardless of positive or negative results, the trial will provide valuable evidence on the appropriate treatment for this stroke population.

References

1. Adams HP, Bendixen BH, Kappelle LJ, Biller J, Love BB, Gordon DL, Marsh EE. Classification of subtype of acute ischemic stroke. Definitions for use in a multicenter clinical trial. TOAST. Trial of Org 10172 in Acute Stroke Treatment.

Stroke. 1993;24:35–41.

2. Wang W, Jiang B, Sun H, Ru X, Sun D, Wang L, Wang L, Jiang Y, Li Y, Wang Y, et al. Prevalence, Incidence, and Mortality of Stroke in China: Results from a Nationwide Population-Based Survey of 480 687 Adults. *Circulation*. 2017;135:759–771.

3. Wu S, Wu B, Liu M, Chen Z, Wang W, Anderson CS, Sandercock P, Wang Y, Huang Y, Cui L, et al. Stroke in China: advances and challenges in epidemiology, prevention, and management. *The Lancet Neurology*. 2019;18:394–405.

4. Fischer U, Baumgartner A, Arnold M, Nedeltchev K, Gralla J, Marco De Marchis G, Kappeler L, Mono M-L, Brekenfeld C, Schroth G, et al. What Is a Minor Stroke? *Stroke*. 2010;41:661–666.

5. Wang Y, Wang Y, Zhao X, Liu L, Wang D, Wang C, Wang C, Li H, Meng X, Cui L, et al. Clopidogrel with Aspirin in Acute Minor Stroke or Transient Ischemic Attack. *N Engl J Med*. 2013;369:11–19.

6. Johnston SC, Easton JD, Farrant M, Barsan W, Conwit RA, Elm JJ, Kim AS, Lindblad AS, Palesch YY. Clopidogrel and Aspirin in Acute Ischemic Stroke and High-Risk TIA. *N Engl J Med*. 2018;379:215–225.

7. Powers WJ, Rabinstein AA, Ackerson T, Adeoye OM, Bambakidis NC, Becker K, Biller J, Brown M, Demaerschalk BM, Hoh B, et al. Guidelines for the Early Management of Patients With Acute Ischemic Stroke: 2019 Update to the 2018 Guidelines for the Early Management of Acute Ischemic Stroke: A Guideline for Healthcare Professionals From the American Heart Association/American Stroke Association. *Stroke* [Internet]. 2019 [cited 2023 Feb 3];50. Available from: https://www.ahajournals.org/doi/10.1161/STR.0000000000000211

8. Khatri P, Tayama D, Cohen G, Lindley RI, Wardlaw JM, Yeatts SD, Broderick JP, Sandercock P. Effect of Intravenous Recombinant Tissue-Type Plasminogen Activator in Patients With Mild Stroke in the Third International Stroke Trial-3: Post Hoc Analysis. *Stroke*. 2015;46:2325–2327.

9. Wong KSL, Wang Y, Leng X, Mao C, Tang J, Bath PMW, Markus HS, Gorelick PB, Liu L, Lin W, et al. Early Dual Versus Mono Antiplatelet Therapy for Acute Non-Cardioembolic Ischemic Stroke or Transient Ischemic Attack: An Updated Systematic Review and Meta-Analysis. *Circulation*. 2013;128:1656–1666.

10. Kim J-T, Park M-S, Choi K-H, Cho K-H, Kim BJ, Park J-M, Kang K, Lee SJ, Kim JG, Cha J-K, et al. Comparative Effectiveness of Dual Antiplatelet Therapy With Aspirin and Clopidogrel Versus Aspirin Monotherapy in Acute, Nonminor Stroke: A Nationwide, Multicenter Registry-Based Study. *Stroke*. 2019;50:3147–3155.

11. Liu L, Wong KSL, Leng X, Pu Y, Wang Y, Jing J, Zou X, Pan Y, Wang A, Meng X, et al. Dual antiplatelet therapy in stroke and ICAS: Subgroup analysis of CHANCE. *Neurology*. 2015;85:1154–1162.

12. Hou X, Li X, Wang X, Chen H. Antiplatelet Therapy in Acute Mild-Moderate Ischemic Stroke (ATAMIS): a parallel, randomised, open-label, multicentre, prospective study. *Stroke Vasc Neurol*. 2018;3:263–267.

13. Kasner SE, Lynn MJ, Chimowitz MI, Frankel MR, Howlett-Smith H, Hertzberg VS, Chaturvedi S, Levine SR, Stern BJ, Benesch CG, et al. Warfarin vs aspirin for symptomatic intracranial stenosis: Subgroup analyses from WASID. *Neurology*. 2006;67:1275–1278.

14. Coutts SB, Hill MD, Campos CR, Choi YB, Subramaniam S, Kosior JC, Demchuk AM. Recurrent Events in Transient Ischemic Attack and Minor Stroke: What Events Are Happening and to Which Patients? *Stroke*. 2008;39:2461–2466.

15. Kim BJ, Park J-M, Kang K, Lee SJ, Ko Y, Kim JG, Cha J-K, Kim D-H, Nah H-W, Han M-K, et al. Case Characteristics, Hyperacute Treatment, and Outcome Information from the Clinical Research Center for Stroke-Fifth Division Registry in South Korea. *J Stroke*. 2015;17:38.

16. Chen Y, Wright N, Guo Y, Turnbull I, Kartsonaki C, Yang L, Bian Z, Pei P, Pan D, Zhang Y, et al. Mortality and recurrent vascular events after first incident

stroke: a 9-year community-based study of 0·5 million Chinese adults. *The Lancet Global Health*. 2020;8:e580–e590.

17. Wang P, Wang Y, Zhao X, Du W, Wang A, Liu G, Liu L, Ji R, Wang C, Dong K, et al. In-hospital medical complications associated with stroke recurrence after initial ischemic stroke: A prospective cohort study from the China National Stroke Registry. *Medicine*. 2016;95:e4929.

18. An International Randomized Trial Comparing Four Thrombolytic Strategies for Acute Myocardial Infarction. *N Engl J Med*. 1993;329:673–682.

19. Hachinski V. World Stroke Day 2008: "Little Strokes, Big Trouble." *Stroke*. 2008;39:2407–2408.

20. Ge F, Lin H, Liu Y, Li M, Guo R, Ruan Z, Chang T. Dual antiplatelet therapy after stroke or transient ischaemic attack - how long to treat? The duration of aspirin plus clopidogrel in stroke or transient ischaemic attack: a systematic review and meta-analysis. *Eur J Neurol*. 2016;23:1051–1057.

21. Chi N, Wen C, Liu C, Li J, Jeng J, Chen C, Lien L, Lin C, Sun Y, Chang W, et al. Comparison Between Aspirin and Clopidogrel in Secondary Stroke Prevention Based on Real-World Data. *JAHA*. 2018;7:e009856.

22. Wong KSL, Chen C, Fu J, Chang HM, Suwanwela NC, Huang YN, Han Z, Tan KS, Ratanakorn D, Chollate P, et al. Clopidogrel plus aspirin versus aspirin alone for reducing embolisation in patients with acute symptomatic cerebral or carotid artery stenosis (CLAIR study): a randomised, open-label, blinded-endpoint trial. *The Lancet Neurology*. 2010;9:489–497.
