# Supplementary material for: Minor stroke patients with mild-moderate diastolic blood pressure derive greater benefit from dual antiplatelet therapy
Source: Hypertens Res. 2023 Sep 5;47(2):291–301. doi: 10.1038/s41440-023-01422-8 (PMC10838769; doi:10.1038/s41440-023-01422-8)
Supplement: Supplementary file 3 — STROBE Statement [file 41440_2023_1422_MOESM3_ESM.pdf]

STROBE Statement—checklist of items that should be included in reports of observational studies

	Item No.	Recommendation	Page No.	Relevant text from manuscript
**Title and abstract**	1	(*a*) Indicate the study's design with a commonly used term in the title or the abstract	Title page	
		(*b*) Provide in the abstract an informative and balanced summary of what was done and what was found	Page 1	Line 1-21
**Introduction**				
Background/rationale	2	Explain the scientific background and rationale for the investigation being reported	Page 2-3	Line 24-53
Objectives	3	State specific objectives, including any prespecified hypotheses	Page 3	Line 53-54
**Methods**				
Study design	4	Present key elements of study design early in the paper	Page 3	Line 58-60
Setting	5	Describe the setting, locations, and relevant dates, including periods of recruitment, exposure, follow-up, and data collection	Page 3	Line 59-60, 62, 72-74, 108-109
Participants	6	(*a*) *Cohort study*—Give the eligibility criteria, and the sources and methods of selection of participants. Describe methods of follow-up<br>*Case-control study*—Give the eligibility criteria, and the sources and methods of case ascertainment and control selection. Give the rationale for the choice of cases and controls<br>*Cross-sectional study*—Give the eligibility criteria, and the sources and methods of selection of participants	Page 3	Line 62-69, 108-110
		(*b*) *Cohort study*—For matched studies, give matching criteria and number of exposed and unexposed<br>*Case-control study*—For matched studies, give matching criteria and the number of controls per case	-	-
Variables	7	Clearly define all outcomes, exposures, predictors, potential confounders, and effect modifiers. Give diagnostic criteria, if applicable	Page 4-5	Line 71-110
Data sources/ measurement	8*	For each variable of interest, give sources of data and details of methods of assessment (measurement). Describe comparability of assessment methods if there is more than one group	Page 4-5	Line 88-97
Bias	9	Describe any efforts to address potential sources of bias	Page 6	Line 125-127
Study size	10	Explain how the study size was arrived at	Protocol page 4	Line 113-120

Continued on next page

Quantitative variables	11	Explain how quantitative variables were handled in the analyses. If applicable, describe which groupings were chosen and why	Page 6	Line 113-117
Statistical methods	12	(*a*) Describe all statistical methods, including those used to control for confounding	Page 6	Line 112-130
		(*b*) Describe any methods used to examine subgroups and interactions	Page 6	Line 116-119
		(*c*) Explain how missing data were addressed	Protocol page 5	Line 151-153
		(*d*) *Cohort study*—If applicable, explain how loss to follow-up was addressed<br>*Case-control study*—If applicable, explain how matching of cases and controls was addressed<br>*Cross-sectional study*—If applicable, describe analytical methods taking account of sampling strategy	Page 7	Line 134-135
		(*e*) Describe any sensitivity analyses	Page 9-10	Line 195-204
**Results**				
Participants	13*	(a) Report numbers of individuals at each stage of study—eg numbers potentially eligible, examined for eligibility, confirmed eligible, included in the study, completing follow-up, and analysed	Page 7	Line 134-136
		(b) Give reasons for non-participation at each stage	Page 7	Line 134-136
		(c) Consider use of a flow diagram	Page 7	Line 134-135
Descriptive data	14*	(a) Give characteristics of study participants (eg demographic, clinical, social) and information on exposures and potential confounders	Page 7	Line 135-145
		(b) Indicate number of participants with missing data for each variable of interest	Protocol page 5	Line 151-153
		(c) *Cohort study*—Summarise follow-up time (eg, average and total amount)	Page 5	Line 103
Outcome data	15*	*Cohort study*—Report numbers of outcome events or summary measures over time	Page 7-9	Line 148-152, 183-191
		Case-control study—Report numbers in each exposure category, or summary measures of exposure	-	-
		Cross-sectional study—Report numbers of outcome events or summary measures	-	-
Main results	16	(*a*) Give unadjusted estimates and, if applicable, confounder-adjusted estimates and their precision (eg, 95% confidence interval). Make clear which confounders were adjusted for and why they were included	Page 7-9	Line 152-159, 164-180, 191-193
		(*b*) Report category boundaries when continuous variables were categorized	Page 5	Line 90-92
		(*c*) If relevant, consider translating estimates of relative risk into absolute risk for a meaningful time period	-	-

Continued on next page

Other analyses	17	Report other analyses done—eg analyses of subgroups and interactions, and sensitivity analyses	Page 9-10	Line 195-204
**Discussion**				
Key results	18	Summarise key results with reference to study objectives	Page 10	Line 207-209
Limitations	19	Discuss limitations of the study, taking into account sources of potential bias or imprecision. Discuss both direction and magnitude of any potential bias	Page 13	Line 273-279
Interpretation	20	Give a cautious overall interpretation of results considering objectives, limitations, multiplicity of analyses, results from similar studies, and other relevant evidence	Page 10-13	Line 211-271
Generalisability	21	Discuss the generalisability (external validity) of the study results	Page 12-13	Line 246-248, 269-271
**Other information**				
Funding	22	Give the source of funding and the role of the funders for the present study and, if applicable, for the original study on which the present article is based	Page 14	Line 294-295

*Give information separately for cases and controls in case-control studies and, if applicable, for exposed and unexposed groups in cohort and cross-sectional studies.

Note: An Explanation and Elaboration article discusses each checklist item and gives methodological background and published examples of transparent reporting. The STROBE checklist is best used in conjunction with this article (freely available on the Web sites of PLoS Medicine at http://www.plosmedicine.org/, Annals of Internal Medicine at http://www.annals.org/, and Epidemiology at http://www.epidem.com/). Information on the STROBE Initiative is available at www.strobe-statement.org.
